# Targeted Inhibition of Anti-Inflammatory Regulator Nrf2 Results in Breast Cancer Retardation In Vitro and In Vivo

**DOI:** 10.3390/biomedicines9091119

**Published:** 2021-08-30

**Authors:** Venugopal R. Bovilla, Mahadevaswamy G. Kuruburu, Vidya G. Bettada, Jayashree Krishnamurthy, Olga A. Sukocheva, Rajesh K. Thimmulappa, Nanjunda Swamy Shivananju, Janardhan P. Balakrishna, SubbaRao V. Madhunapantula

**Affiliations:** 1 Department of Biochemistry (DST-FIST Supported Department), JSS Medical College, JSS Academy of Higher Education & Research, Mysore 570015, Karnataka, India; venugopal.reddy@jssuni.edu.in (V.R.B.); mahadevaswamykg@jssuni.edu.in (M.G.K.); vidyabg@jssuni.edu.in (V.G.B.); rajeshkt@jssuni.edu.in (R.K.T.); 2 Center of Excellence in Molecular Biology and Regenerative Medicine (CEMR) Laboratory (DST-FIST Supported Center), JSS Medical College, JSS Academy of Higher Education & Research, Mysore 570015, Karnataka, India; 3 Public Health Research Institute of India (PHRII), Mysuru 570020, Karnataka, India; 4 Department of Pathology, JSS Medical College, JSS Academy of Higher Education & Research, Mysore 570015, Karnataka, India; kjayashree@jssuni.edu.in; 5 College of Nursing and Health Sciences, Flinders University, Bedford Park, SA 5042, Australia; 6 Department of Biotechnology, JSS Technical Institutions Campus, JSS Science and Technology University, Mysore 570006, Karnataka, India; nanjundaswamy@sjce.ac.in; 7 Department of Stem Cell Biology, Stellixer Biotech Pvt Ltd., Banglore 560058, Karnataka, India; janardhan@stellixir.com; 8 Leader, Special Interest Group in Cancer Biology and Cancer Stem Cells (SIG-CBCSC), JSS Academy of Higher Education & Research, Mysore 570015, Karnataka, India

**Keywords:** breast cancer, Nrf2, brusatol, Ehrlich Ascites Carcinoma cells, chemo sensitization, tumorigenesis

## Abstract

Nuclear factor erythroid-2 related factor-2 (Nrf2) is an oxidative stress-response transcriptional activator that promotes carcinogenesis through metabolic reprogramming, tumor promoting inflammation, and therapeutic resistance. However, the extension of Nrf2 expression and its involvement in regulation of breast cancer (BC) responses to chemotherapy remain largely unclear. This study determined the expression of Nrf2 in BC tissues (n = 46) and cell lines (MDA-MB-453, MCF-7, MDA-MB-231, MDA-MB-468) with diverse phenotypes. Immunohistochemical (IHC)analysis indicated lower Nrf2 expression in normal breast tissues, compared to BC samples, although the difference was not found to be significant. However, pharmacological inhibition and siRNA-induced downregulation of Nrf2 were marked by decreased activity of NADPH quinone oxidoreductase 1 (NQO1), a direct target of Nrf2. Silenced or inhibited Nrf2 signaling resulted in reduced BC proliferation and migration, cell cycle arrest, activation of apoptosis, and sensitization of BC cells to cisplatin in vitro. Ehrlich Ascites Carcinoma (EAC) cells demonstrated elevated levels of Nrf2 and were further tested in experimental mouse models in vivo. Intraperitoneal administration of pharmacological Nrf2 inhibitor brusatol slowed tumor cell growth. Brusatol increased lymphocyte trafficking towards engrafted tumor tissue in vivo, suggesting activation of anti-cancer effects in tumor microenvironment. Further large-scale BC testing is needed to confirm Nrf2 marker and therapeutic capacities for chemo sensitization in drug resistant and advanced tumors.

## 1. Introduction

Despite the therapeutic progress and introduction of various public health programs, breast cancer (BC) incidence and mortality rates continue to increase [1]. Current BC clinical interventions include a large variety of surgical procedures, chemotherapeutic agents, hormonal therapy, radiotherapy, and immunotherapeutic approaches [2,3,4,5]. However, the highly successful BC chemotherapeutic agents, including doxorubicin/epirubicin, docetaxel, and paclitaxel [6,7], target early-stage tumors. Advanced stage BCs are managed by carboplatin, cisplatin, gemcitabine, capecitabine, vinorelbine, abraxane [8,9,10], or combined therapies [11,12]. Despite reasonable success, extensive and prolonged treatment often results in the development of BC resistance [3], accompanied by systemic toxicity [13]. The benefits of advanced genetic testing and personalized medicine-based approaches for BC treatment are not yet available for the wider population, especially in developing countries [14,15,16]. Therefore, identifying multi-functional key regulatory proteins that can be effectively targeted to control malignant transformation, spreading, and therapy resistance, has become increasingly important.

Nuclear factor erythroid-2 related factor-2 (Nrf2) is a transcription factor that directs host responses to inflammation and intracellular oxidative stress via modulation of a diverse group of effectors. During induction of oxidative burst and anti-inflammatory signaling, Nrf2 translocates into the nucleus, binds to electrophile response element (EpRE), and triggers EpRE-mediated transcription of various genes with an antioxidant response element (ARE)-containing promoter region [17,18]. Among those genes are cytoprotective NADPH quinone oxidoreductase 1 (NQO1), superoxide dismutase (SOD), glutathione S-transferase (GST), heme oxygenase 1 (HO1), and hypoxia inducible factor 1-α (HIF1α) [19,20]. Nrf2 is known to protect normal cells from oxidative stress and inflammation-induced cellular damage. Unfortunately, the elevated Nrf2 also protects cancer cells from chemotherapeutic- and radiation-induced damage [21,22,23]. Nrf2-regulated antioxidant response was suggested to play a crucial role in controlling BC cell survival [24] and development of drug resistance [25].

The diversity of Nrf2-activated network is mediated by potential interaction of different Nrf2 domains with transcription coactivators, including CHD6 (chromo-ATPase/helicase DNA-binding protein), CBP (cAMP-response-element-binding protein (CREBP)-binding protein) [11], and the nuclear cofactor RAC3/AIB1/SRC-3 [26]. Complex and often contradictory relationships were demonstrated for Nrf2 and NF-κB signaling network (the leading cancer-related intracellular effector) linked to various cellular responses to stress, inflammation, and oncogenesis [27,28]. For instance, Nrf2 and NF-κB were shown to compete for CBP and other co-activators during resolution of inflammation and cancer progression [17,27]. During cancer treatment with bortezomib, both signaling pathways promoted chemoresistance in acute myeloid leukemia cells [28]. Accordingly, targeted inhibition of Nrf2 using siRNA or pharmacological agents was shown to inhibit the development of lung, colon, and rectal carcinomas [29,30]. In BCs, elevated levels of Nrf2 trigger proliferation and metastatic behavior via activation of the RhoA/ROCK pathway and G6PD/HIF1α/Notch signaling cascades [21]. Increased Nrf2 expression stimulates anabolic pathways and tumor-promoting inflammation in BCs [31]. Estrogen receptor positive (ER+) but human epidermal growth factor receptor 2 negative (HER2-) BCs expressing high levels of Nrf2 are more resistant to treatments compared to those tumors with very low Nrf2 [21,32]. However, it remains to be confirmed whether the expressed Nrf2 is functionally active in BCs. Furthermore, Nrf2 inhibition as a method to sensitize BC cells to chemotherapeutic agents has not been sufficiently explored. Nrf2 can be inhibited by various synthetic and naturally occurring pharmacological agents, including ML385, AEM1, brusatol, apigenin, trigonelline, berberine, and parthenolide [33].

Brusatol is a natural compound (quassinoid) isolated from *Brucea javanica*. Anti-cancer properties of the compound and its ability to inhibit Nrf2 signaling were demonstrated in different malignancies [30,33,34]. However, specific molecular targets of brusatol and associated in vivo pharmacological and systemic effects of the agent remain largely unclear. Recent study reported brusatol-activated increases in the intrinsic metabolic burden in cancer cells, thus, making this agent increasingly attractive for future drug development [35]. Several studies have shown anti-proliferative capacity of brusatol in cancers that were mediated by deregulated Nrf2 signaling [29,36,37]. Despite the observed pro-apoptotic effects, majority of Nrf2 inhibitors were shown to have only modest success in clinical trials [38], suggesting that Nrf2 is associated with a complex mechanism of signaling which remains unexplored. Therefore, we used Brusatol to inhibit Nrf2 in BC cell lines and aimed to clarify the systemic effects of this agent in vivo.

Accordingly, Nrf2 activation of its downstream signaling effectors in BC cell lines was addressed in this study. Although Nrf2 was linked to BC progression, the question of whether Nrf2 can be exploited as a key therapeutic target to inhibit BC growth remains uncertain. This study aimed to test the levels of Nrf2 expression and cell responses to Nrf2 inhibition in a set of BC tissues and cell lines. The expression pattern of Nrf2 was assessed in grade-II and grade-III BCs and compared to proximal normal tissues. Ehrlich Ascites Carcinoma (EAC) cells were found to have a significantly increased level of Nrf2. Using a preclinical animal model, the current study investigated whether grafted EAC cells are suitable for testing the anti-cancer efficacy of Nrf2 inhibitors. A significant reduction in grafted tumor size was observed upon the administration of brusatol in Swiss albino mice, indicating that Nrf2 inhibition could be a potentially viable anti-BC strategy. Inhibition of Nrf2 signaling in combination with an established chemotherapeutic agent cisplatin was also tested to assess the suitability of combination strategies for effective tumor inhibition.

## 2. Materials and Methods

### 2.1. Collection of Tumor Samples and Used Chemicals

The current study was approved by the Institutional Ethics Committee, which allowed collecting tumor and normal breast samples from BC patients (approval no. JSSMC/IEC/14/1991/2017–2018, dated 5 June 2017). The patients visited JSS Hospital and were referred for pathological examinations. The consent approved using the collected tissues for research purposes. Patient tumors and normal tissues were paraffin-embedded at the Department of Pathology, JSS Medical College, JSS Academy of Higher Education & Research, Mysore, Karnataka.

BC cell lines MCF-7 (passage #40–55), T47D (passage #60–75), MDA-MB 453 (passage #70–90), MDA-MB-231(passage #30–50), MDA-MB-468 (passage #50–65), and lung cancer cell line A549 (passage #30–50) were procured from National Center for Cell Science, Pune, Maharashtra, India. Normal lung epithelial cell line BEAS-2B (passage #20–40) was provided by Dr. Rajeshkumar Thimmulappa (JSS Medical College, JSS AHER, Mysore, Karnataka). EAC cells were provided by Dr. Prabhakar B.T (Molecular Biomedicine Laboratory, Post Graduate Department of Studies and Research in Biotechnology Sahyadri Science College Kuvempu University, Shimoga, Karnataka, India). Cell line phenotypes were confirmed and characterized annually for morphological and cell line specific markers as described previously [39].

Primary antibodies for Nrf2 (cat#: ab62352), NQO1 (cat#: 62262), were obtained from Abcam (Cambridge, MA, USA) and Cell Signaling Technologies (Danvers, MA, USA). Secondary antibodies (Rabbit cat#: SC2357 and Goat cat#:SC2020) and α-Enolase (cat#:SC-7455) were from Santa Cruz Biotechnology (Santa Cruz, CA, USA). Ki67 (cat#: PM210) and CD31 (cat#: PR021) were from PathnSitu Biotechnologies Pvt Ltd., (Secunderabad, Telangana State, India). SiRNA to Nrf2 (HSS 181506, HSS 181505, HSS107130) and lipofectamine RNAiMAX (cat#:13778150), FBS (cat#:10270106), Pen-Strep (cat#:150763), cDNA Reverse Transcription Kit (cat#: 4388950), DyNAmo Flash SYBR Green qPCR Kit (cat#: F-415L), and TRIzol (cat#:15596026) reagents were from Thermo Fisher Scientific (Waltham, MA, USA).

Pharmacological agents brusatol (cat#:SML 1868), diallyl disulfide (DADS) (cat#: SMB00378), dicoumarol (cat#: M1390-5G), cell culture grade DMSO (cat#: D2650), glucose-6-phosphate (G6P) dehydrogenase (G6PDH) (cat#: 10165875001), 3-(4,5-dimethylthiazol-2-yl)-2,5-diphenyltetrazolium bromide tetrazolium (MTT) (cat#: M5655), menadione (cat#: M9429), NP40 (cat#: 492016), radioimmunoprecipitation assay (RIPA) buffer (cat#: R0278), protease inhibitor cocktail (cat#: S8820), G6P (cat#:G7250), nicotinamide adenine dinucleotide phosphate (NADP) (cat#: N5755), flavin adenine dinucleotide (FAD) (cat#: F6625), bovine serum albumin (BSA) (cat#: 05479), camptothecin (cat#: 390238), itraconazole (cat#: I6657), and cisplatin (cat#:1134357) were from Sigma Chemical Company (St. Louis, MO, USA). All cell culture plastics were from Techno Plastic Products (TPP) Pvt Ltd. (Bengaluru, Karnataka, India). Cell culture media Dulbecco’s Modified Eagle Medium (DMEM) with high glucose (4.5g/L) (cat#: AL111), trypsin-EDTA (0.25%) (cat#: T001), Dulbecco’s phosphate buffered saline (DPBS) (cat#: TL1OO6), ciprofloxacin (cat#: A032), acridine orange (AO, cat#: TC262), and ethidium bromide (EtBr, cat#: MB071) were from HiMedia Laboratories Pvt Ltd. (Bengaluru, Karnataka, India). Swiss albino mice were purchased from Biogen Laboratory Animal Facility (Bengaluru, Karnataka, India).

### 2.2. Collection of Breast Cancer Tissues, Construction of a Tissue Array, and Immunohistochemistry (IHC)

Breast tissues were collected, fixed, paraffin-embedded, and stained using Hematoxylin and Eosin (H&E). The tissue sections were screened and representative malignant areas were identified by an experienced pathologist. The corresponding areas in the paraffin blocks were marked and tissue cores of about 4 mm in size were punched out using a tissue punch. Tissue array (TA) blocks were constructed manually in rows and columns by incorporating tissue cores (Supplementary Figure S1). Sections from the TA blocks were stained using H&E and processed for immunohistochemical (IHC) analysis to measure Nrf2 expression as described previously [40].

To assess Nrf2 expression, the selected slides were deparaffinized and antigens were retrieved by dipping in an antigen retrieval buffer (10 mM sodium citrate, pH 6.0). Hydrogen peroxide (3%) was applied for 10 min to quench the endogenous peroxidases. The sections were incubated with primary anti-Nrf2 antibodies (1:200 dilution i.e., 5 µg/mL) for 12 h at 4 °C and washed with TBS (3 times, 5 min each). Tumor tissues were stained using antibodies for Ki67 (for proliferation), CD31 (for vessel density), and NQO1 (oxidative stress marker). The sections were incubated with secondary antibody conjugated with horseradish peroxidase (HRP) for 30 min at room temperature; and washed with TBS (3 times, 5 min each). The bound antibodies were visualized using a 3,3′-diaminobenzidine (DAB)-chromogen substrate (50 µL of DAB Chromogen in 1.0 mL of DAB buffer). The sections were rinsed in running water and counter stained with hematoxylin. Excess hematoxylin was removed by rinsing in water for 5 min.

The H&E stained and IHC processed slides were assessed under a light microscope and photographed to determine the tissue morphology and level of cancer marker expression. Representative photomicrographs were captured using Olympus (BX 53, Olympus Corporation Shinjuku, Tokyo, Japan) microscope operating with 40× magnifying lenses. The expression grading was conducted by two experienced pathologists who independently counted the number of positively stained cells (represented as percentage of stained cells in the field/per slide) and estimated the intensity of staining. The staining intensity was graded as 0 (no staining), 1+ (weakly stained), 2+ (moderately stained), and 3+ (strongly stained) [41].

### 2.3. Isolation, Estimation of Total Protein, and Western Blotting Analysis

To isolate total proteins from tumor and normal tissues, the samples were washed with PBS, and chopped into smaller pieces. The chopped tissue was snap frozen in liquid nitrogen, powdered, and homogenized in pre-chilled lysis buffer (0.8% NP40 and 2 mM EDTA) solution containing protease inhibitor cocktail mix. The solution was sonicated for 1.0 min (PCI Analytics, Mumbai, Maharashtra, India), centrifuged at 21,000× *g* for 30 min at 4 °C. Protein content was estimated using bicinchoninic acid (BCA) method with BSA standards. Contrary to tissue homogenates, the protein lysates were harvested from cell cultures using lysis buffer (50 mM HEPES (pH 7.5), 150 mM NaCl, 10 mM EDTA, 10% glycerol, 1% Triton X-100, 1 mM sodium orthovanadate, 0.1 mM sodium molybdate, 1 mM phenylmethylsulfonyl fluoride (PMSF), 20 g/mL aprotinin, and 5 g/mL leupeptin according to a procedure described previously [42]. The total protein content was estimated using a commercially available BCA kit from Pierce (Thermo Fisher Scientific, Rockford, IL, USA).

Expression of Nrf2 was measured by western blotting as described previously [43]. Total protein (50 µg) was loaded per lane onto SDS-PAGE gels (4–12%, NuPAGE) and subjected to electrophoresis. Blots were probed with antibodies (1:1500 dilution of primary antibody from Abcam and Cell Signaling Technologies, USA and 1:4000 dilution of secondary antibody from Santa Cruz Biotechnology, Santa Cruz, CA, USA). Immunoblots were developed using an enhanced chemiluminescence (ECL) detection system (Thermo Fisher Scientific, Rockford, IL, USA). The intensity of protein bands was quantitated using Image-J software. Intensity of each band was normalized to Enolase loading control; and the data were represented as fold change compared to vehicle treated or normal controls.

### 2.4. Measurement of NQO1 Activity

NQO1 activity was measured using standard reaction of glucose-6-phosphate (G6P) incubation with G6P dehydrogenase (G6Pdase) that produced NADPH, which, in turn, was used to reduce menadione into menadiol [44]. The level of blue colored formazan was measured at 610 nm using a multimode plate reader. Dicoumarol was used to measure the background activity contributed by other reductases. Aliquots of total protein (10 µg of a protein in a total volume of 40 µL) were incubated with 200 µL of NQO1 cocktail with or without dicoumarol (three with and three without dicoumarol; six wells for each test). The absorbance was read at 610 nm for a period of 30 min with 1 min interval. NQO1 activity was calculated by subtracting the readings of samples with inhibitor from the ones without the inhibitor. Following this, the optical density (OD) value per minute was calculated and mole units were determined by multiplying the OD/minute/molar extinction coefficient of MTT (11,300 M^−1^ cm) with protein concentration of a sample [43]. The NQO1 activity was expressed as µmol/min/mg protein. The method was also used to assess NQO1 activity in cell lines.

### 2.5. siRNA Mediated Nrf2 Knockdown Using Lipofectamine RNAi Max Reagent

Expression of Nrf2 was transiently inhibited by transfecting cells with siRNA (from Thermo-Fisher Scientific, Waltham, MA, USA) using a reverse transfection protocol. Before transfection, cells were grown in a medium without antibiotics and harvested using trypsin. The transfection was conducted according to the manufacturer instructions. The collected RNAs and protein were analyzed using quantitative real-time PCR (qRT-PCR) and western blotting, respectively. To assess the knockdown effect on cell viability, 1 × 10^4^ control and transfected cells/well in 100 µL media were plated and allowed to grow for 48 and 72 h. Cell viability was determined using MTS (3-(4,5-dimethylthiazol-2-yl)-5-(3-carboxymethoxyphenyl)-2-(4-sulfophenyl)-2H-tetrazolium))assay (cat#: G111A, Promega, Madison, WI, USA).

### 2.6. Analysis of Gene Expression Using qRT-PCR and Gel Electrophoresis of Total RNA

Quantification of gene expression was carried out using qRT-PCR as described previously [45]. RNA was isolated according to the standard procedure [46] using the TRIzol reagent. The RNA quality and quantity were estimated using a spectrophotometer. Additionally, the quality of RNA was determined using 1% bleach gel electrophoresis as described previously [47]. The samples were stored at −80 °C for further usage. Quality of isolated RNA was measured using bleach gel electrophoresis. The bleach gel (1%) was prepared by mixing 1 g of agarose in 100 mL 1X TAE buffer (40 mM Tris (pH 7.6), 20 mM acetic acid, and 1 mM EDTA), containing 600.0 µL 5% sodium hypochlorite. The samples were mixed with 6X loading dye (30% glycerol and 0.25% (*W*/*V*) bromophenol blue) and loaded into gel-loaded cassette. Samples (24 µL) were separated at 100 volts (1–5 volts/cm) during 60 min. The separated RNA bands were visualized using UV light in a UV-transilluminator (Syngene G-BOX XR5, Syngene, Frederick, MD, USA). Appearances of 28S and 18S RNA bands at a ratio of 2:1 indicated the presence of intact RNA.

Reverse transcription reaction was performed using High-Capacity cDNA Reverse Transcription Kit. The final sample volume of 20.0 µL contained 1000 ng of total RNA, 100 ng of random hexamers, 2.0 µL reverse transcription buffer, 2.5 mM MgCl_2_, 1 mM dNTP, 20 U of superscript reverse transcriptase, and nuclease free diethyl pyrocarbonate (DEPC)-treated water as detailed in High-Capacity cDNA Reverse Transcription kit protocol. The reaction steps and conditions were as follows: Step 1: 25 °C for 10 min; Step 2: 37 °C for 120 min; Step 3: 85 °C for 5 min; Step 4: 4 °C. After completion of cDNA synthesis, 80 µL nuclease free water was added to vials (final concentration of the cDNA was 10 ng/µL) and samples were stored at 4 °C.

Human *Nrf2*, its target gene *NQO1*, and the house keeping control gene *GAPDH* were quantified using specific primers and DyNAmo Color Flash SYBR Green QPCR Kit (Finnzymes) according to the manufacturer’s instruction. Reaction mixture composition (for 20 µL) was as following: (1) SYBR green dye −10 µL; (2) primers: forward −0.2 µM and reverse −0.2 µM (Table 1); (3) template: 50 ng; (4) nuclease free water. All primers were purchased from Sigma (Bengaluru, Karnataka, India). Quantitative RT-PCR experiment was performed using Qiagen Rotor Gene-Q (Qiagen Rotor Gene-Q-5 Plex, Qiagen, Antwerp, Belgium). Relative fold change was calculated using 2^-ΔΔct^ method as described previously [45]; ΔΔCT = (CT of gene of interest in TEST sample—CT of internal control of test sample)—(CT gene of interest in CONTROL sample—CT internal control of control sample).

### 2.7. Cytotoxic Potential of Nrf2 Inhibitor Brusatol

The anti-cancer activity of brusatol was measured as described previously [55]. Various BC cells (MCF-7, MDA-MB-231, or MDA-MB-468) (1.0 × 10^4^ cells in 100 µL DMEM with 10% FBS) were seeded in 96-well plates and cultured in an incubator at 37 °C with 5% CO_2_ and 90% relative humidity. Following this, when cell confluence reached about 60 to 70% (36 h later), the cells were exposed to gradual concentrations of brusatol (ranging from 0.31 to 10 µM) for 24 or 48 h. Following this treatment, cell viability was measured using sulforhodamine B (SRB) and MTT(3-(4,5-Dimethylthiazol-2-yl)-2,5-Diphenyltetrazolium Bromide) assays [56,57]. Diallyl disulfide (DADS) (1 mM) was used as positive control.

### 2.8. Cell Cycle Analysis Using Propidium Iodide (PI) Staining

Cell cycle analysis was carried out as described previously [58]. MDA-MB-468 or MCF-7 cells (3 × 10^5^ in 2 mL medium) were cultured in 6-well plates for 24 h in a CO_2_ incubator at 37 °C. Moreover, 60–70% confluent cells were washed with PBS and exposed to gradually increasing concentrations of brusatol for 48 h. Camptothecin (25 µM) was used as positive control. DMSO (0.1%) served as vehicle control. Cells grown in medium with no treatments served as untreated control. Following 48 h incubation, the medium was removed. The cells were washed with PBS, trypsinized, and collected into a 15 mL polystyrene tube. The collected cells were centrifuged (5 min at 300× *g* at 25 °C) and the medium was discarded. The cell pellet was washed with PBS and fixed using 4 mL of cold 70% ethanol, and the fixed cells were centrifuged at ~500× *g* for 5 min. The pelleted cells were washed twice with PBS.

To ensure that only DNA is stained, the cells were treated with 5 μg/mL RNase (50 µL) and the cells were incubated with PI (400 μL PI solution per million cells) for 10 min at room temperature. The stained cells were analyzed using flow cytometry (BD FACSCalibur, model: 343202-FACSCALIBUR 4 CLR, BD Biosciences, San Jose, CA, USA) with three filters (GFP-515/15, YFP-540/20BP, RFP—610/20BP). Cell quest pro v.6.0 software was used for cell analysis.

### 2.9. Assessment of Cell Migration Using Scratch Assay

Scratch assay was carried out as described previously [59,60]. BC cells (1 × 10^5^ cells/well) were seeded in 12-well plates and incubated in a CO_2_ incubator until cells reached 80% confluence. Following this, a linear wound (~0.7 mm width) was produced using a sterile pipette tip (10 µL). Then, loosely bound cancer cells were washed carefully with PBS and the cells were treated with brusatol (19, 78, 312, or 1250 nM), or a positive control itraconazole (5 µg/mL), or vehicle control [61]. Photomicrographs were captured at 0, 24, 48, and 72 h. The scratch area was calculated using Image-J software. To exclude the impact of proliferation (when growing in 10% FBS medium) on gap closure, the BC cells were grown in DMEM supplemented with 1% FBS and the scratch assay carried out as detailed elsewhere [62,63].

### 2.10. Apoptosis Assay with Acridine Orange (AO) and Ethidium Bromide (EtBr)

To determine cell death related changes, an AO and EtBr staining method was adopted. Control and pre-treated cells (0.5 × 10^6^) were trypsinized and mixed gently to obtain a single cell suspension. Trypsin was neutralized by the addition of complete medium, and the cell suspension was centrifuged at 900× *g* for 5 min. The cell pellet was resuspended in 20 μL PBS. The cell suspension was incubated with 10 μL EtBr (100 μg/mL in PBS) and 10 μL AO (100 μg/mL in PBS) mixture for 10 min. The stained cells were assessed using an Olympus fluorescence microscope (BX 53, Olympus Corporation Shinjuku, Tokyo, Japan) operating with TRITC and FITC filters. All images were captured using green and red channels, and subsequently merged to visualize a combined image showing green (live) and orange/red (apoptotic) cells. At least five different fields were considered for quantification of live and apoptotic cells. The percentage of cells undergoing apoptosis over total cells/field is represented as bar graph.

### 2.11. Assessment of Lymphocytic Infiltration in Solid Tumors

Lymphocytic infiltration was assessed in H&E-stained tumor sections as detailed by [64]. The stained sections were examined using light microscopy at 10× and 40× magnifications for the presence of reactive lymphocytes in tumor and surrounding tissues. The percentage of lymphoid cells surrounding the tumor was scored (1–3), according to the density of lymphocytic infiltration; wherein 1 refers to mild infiltration, 2 refers to moderate infiltration and 3 refers to dense infiltration.

### 2.12. In-Vivo Evaluation of Brusatol Effects in Swiss Albino Mouse Model

Animal experiments were approved by Institutional Animal Ethics Committee from JSS College of Pharmacy, Mysore (approval no. IAEC/JSSCPM/319/2018 4 September, 2018). Swiss albino mice (6–8 weeks old) weighing around 26–30 g was divided into four groups with six animals in each group. Viable EAC cell (5 × 10^6^) suspension was injected into right thigh tissue of six experimental animals as described previously [65]. Six normal control animals were injected with vehicle solution. Volume of developing tumors was measured once in two days using a Vernier Caliper. Beginning from the seventh day after injection, the mice brusatol (0.5 mg/kg and 2 mg/kg body weight) was administered every other day intraperitoneally. The treatment continued for two weeks, and the experiment was terminated on day 23 after injection of tumor cells. Animals were humanely sacrificed, and tumor tissues were collected. The length and width of the tumor was measured and the volume calculated using (W^2^ × L)/2 formula [66]. The collected tumors were washed and processed for further IHC staining analysis. Tumor proteins were also isolated to measure NQO1 activity as described above.

### 2.13. Statistical Analysis

All experiments were repeated three or more times. Data were expressed as mean of three independent experiments ± SD. GraphPad Prism version 6.0 (Graphpad Software, CA, USA) was used for statistical analyses. The results were subjected to one-way ANOVA to compare differences between control and test groups. Tukey’s post hoc test was used as indicated and the “*p*” value of <0.05 was considered significant.

## 3. Results

### 3.1. Diversity of Nrf2 Expression Pattern in BC Tumors and Cell Lines

**Figure 1:**
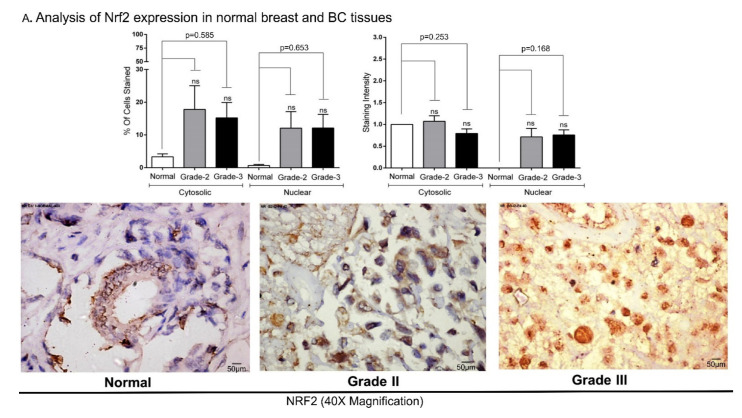

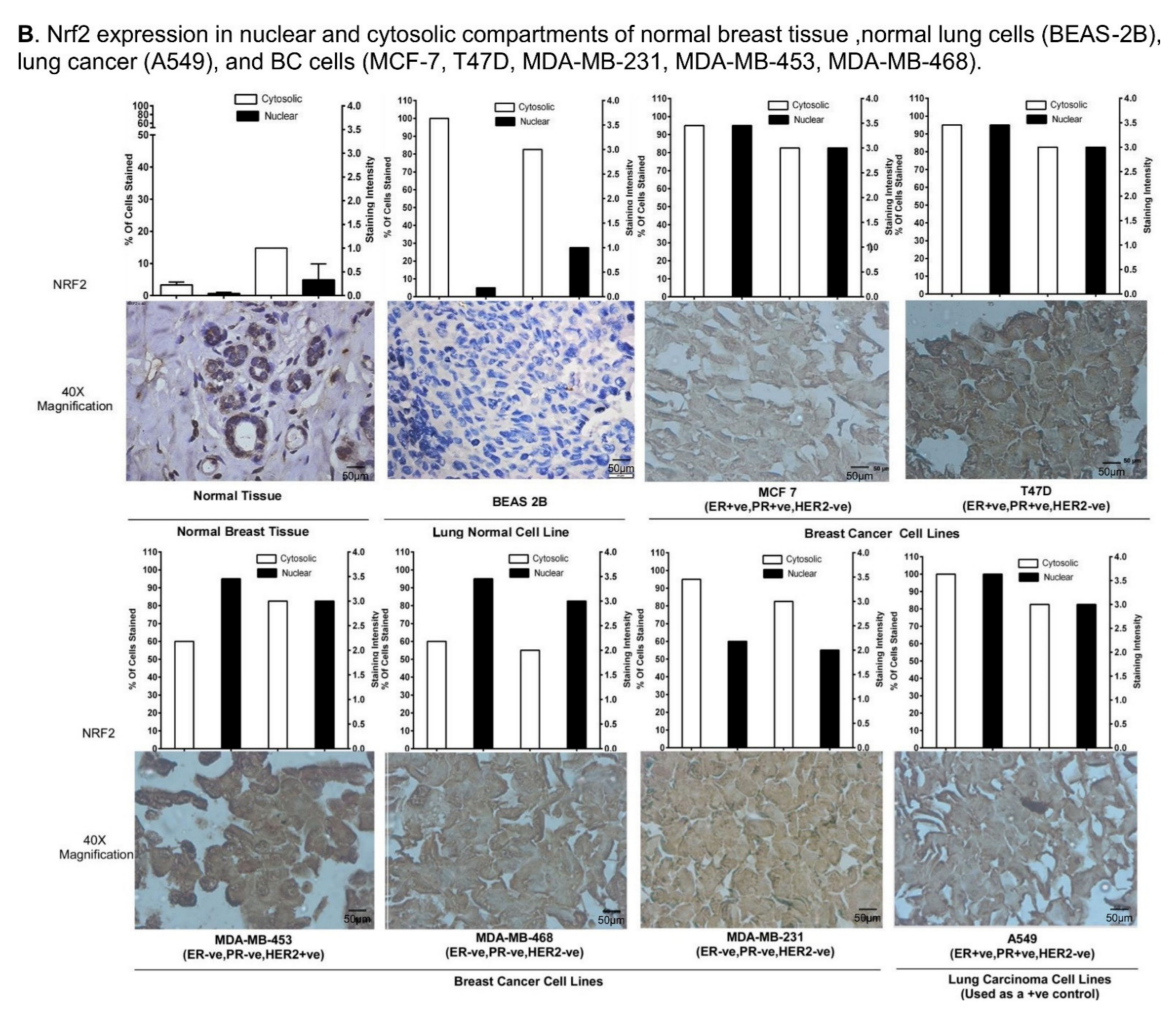
IHC analysis of Nrf2 expression in BC tissues and cell lines: (**A**) Nrf2 expression in normal and grade-II/III BC tissues: The expression of Nrf2 in normal breast tissues (n = 3), grade-II (n = 14), and grade-III (n = 29) BC tissues was analyzed by IHC. Representative microphotographs (40X magnification images) are shown. Please refer to Supplementary Figure S1 for the tissue array preparation procedure. Expression of Nrf2 in cytosolic and nuclear compartments of the same cells/tissues was compared and was not found significantly different. (**B**) Elevated Nrf2 was observed in breast cancer cell lines MCF-7, T47D, MDA-MB-453, MDA-MB-231, MDA-MB-468, and in lung cancer cell line A549. Expression of Nrf2 was visualized using immunocytochemistry (IHC). Irrespective of ER, PR, and HER2 expression status, all BC cell lines and A549 exhibited higher level of Nrf2 compared to normal breast tissue and lung cells. Total cell number was counted for each image. Representative microphotographs (40X magnification, Scale:50 µm) are shown. ns refers to non-significant.

The expression of Nrf2 was measured using immunohistochemistry (IHC) in the tumor tissues collected from grade-II (n = 14) and grade-III (n = 29) BC patients and compared with normal breast tissues (n = 3). Nrf2 staining intensity (scale 0–3) and percentages of stained cells are shown in Figure 1 and Table S1. Analysis of the data showed that Nrf2 expression was lower in normal tissues, although not significant. A relatively low number of normal breast tissue were available for analysis (Figure 1). Less than 3% of normal cells indicated Nrf2 presence, as evidenced by staining in cytosol. Minimal staining was also observed in normal cell nuclei compared to grade-II and grade-III BCs. Nearly 17% of BC cells exhibited staining in both cytosol and nucleus (Figure 1A), although the difference between Nrf2 expression in the cytosol and nucleus compartments was not significant. The IHC staining intensity and number of stained cells were not significantly different between grade-II and grade-III tumors (Figure 1A). All grade-II tumor cells showed positive staining at least in one of the compartments (nucleus or cytoplasm). However, 4 out of 29 grade-III BCs showed no detectable staining for Nrf2 in cytosol and nucleus (Figure 1A). BC patients’ clinical features are shown in Table S3.

To determine variation in the level of Nrf2 in breast cancer cell lines, we prepared cellblocks as described in Supplementary Figure S2. BC cell lines with diverse expression of estrogen receptor (ER), progesterone receptors (PR), and human epidermal growth factor receptor-2 (HER2) were used and included MCF-7 and T47D (ER^+^/PR^+^/HER2^−^), MDA-MB-453 (ER^−^/PR^−^/HER2^+^), MDA-MB-231, and MDA-MB-468 (ER^−^/PR^−^/HER2^−^) cells. A549 (ER^+^/PR^+^/HER2^+^) cells were used as a positive control for Nrf2 expression. A549 cells are marked by loss of heterozygosity (LOH) and functionally inactive Keap1 (a negative regulator of Nrf2) [67]. Normal human BEAS-2B cells were used as a negative control.

IHC analysis indicated that cytosolic Nrf2 was expressed in all cell lines. However, compared to other BC cell lines, MDA-MB-453 and MDA-MB-468 had a lower percentage of cells (only 65%) that stained for Nrf2 in cytosol (Figure 1B). Almost all cell lines were scored “3” for Nrf2 staining intensity of the cytosolic region, with the exception of MDA-MB-468, which scored “2” (moderate staining). The normal lung cell line BEAS-2B exhibited lower intensity staining in the cytosol compared to the nuclear region. Notably, nearly all cell lines exhibited ~100% nuclear Nrf2 expression, except MDA-MB-231 (~60%). Nuclear Nrf2 staining intensity was scored “3” for all cell lines except MDA-MB-231, which scored “2”. Nuclear Nrf2 expression was significantly higher in BC cells compared to normal breast tissue cells. Accordingly, lung cancer cells also demonstrated higher nuclear Nrf2 compared to normal lung epithelial cells BEAS-2B (Figure 1B).

### 3.2. siRNA-Dependent Inhibition of Nrf2 Reduced BC Cells Viability and Sensitized Cells to Cisplatin In Vitro

**Figure 2:**
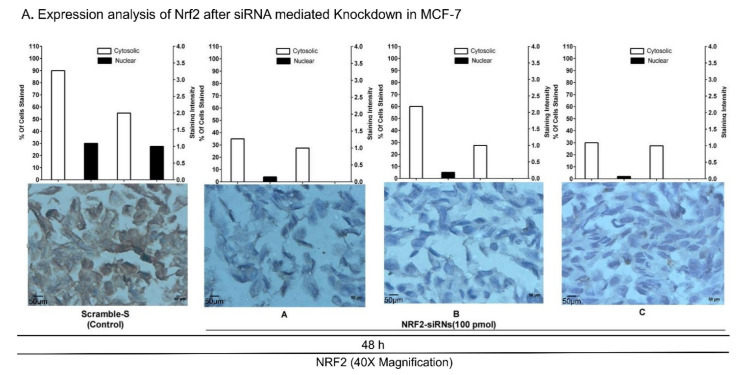

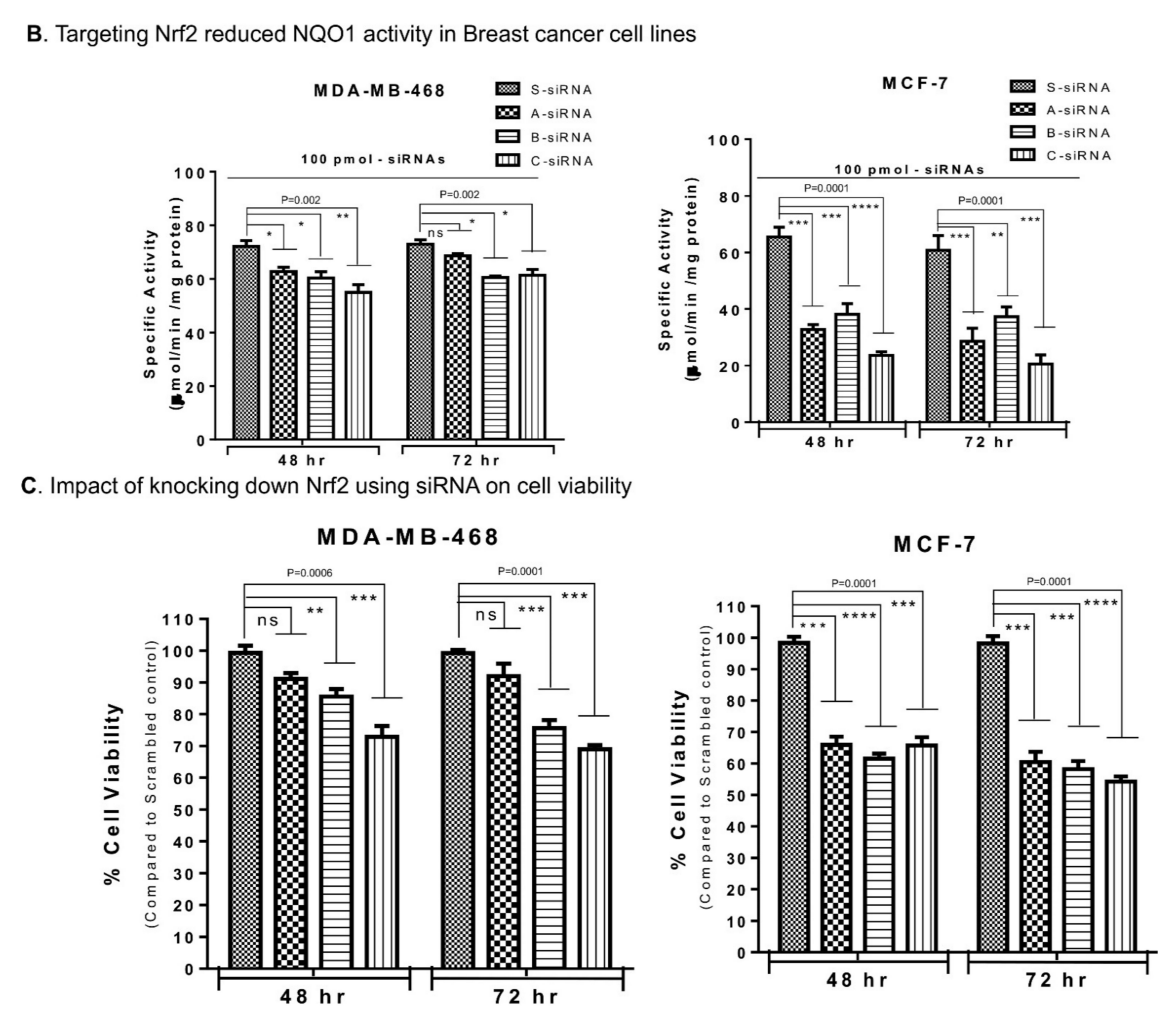
Targeted inhibition of Nrf2 using siRNA in BC cell lines in vitro: (**A**) Nrf2 level was reduced in MCF-7 cells transfected with scrambled siRNA (Control) and three different siRNAs (siNrf2-**A**, siNrf2-**B**, and siNrf2-**C**) (Supplementary Table S2 shows siRNA sequences). Representative images of immunocytochemistry (ICC) slides are shown (40× magnification, Scale: 50 µm). (**B**) Reduced NQO1 activity was observed in MDA-MB-468 and MCF-7 cells (*p* value = 0.002; *p* value = 0.0001) with silenced Nrf2. Data were analyzed using one-way ANOVA. (**C**) Cell viability was determined in MDA-MB-468 and MCF-7 cells using MTS 48 and 72 h after siRNA transfection. A significant reduction in cell growth was observed with siRNA-B and siRNA-C in MDA-MB-468 cell line (*p* value = 0.0006; *p* value = 0.0006). In MCF-7 all the three siRNAs yielded significant inhibition of viability (*p* value = 0.0001; *p* value = 0.0001) (* *p* < 0.05, ** *p* < 0.01, *** *p* < 0.005, **** *p* < 0.001). ns refers to non-significant.

To silence Nrf2 expression in BC cell lines, 3 siRNAs (siNrf2-A, siNrf2-B, and siNrf2-C) for different Nrf2 regions were introduced to BC cells using lipofectamine RNAiMAX reagent (Table S2) [68]. The efficacy of transfection was above 90%. Knockdown of Nrf2 was confirmed using qRT-PCR (4- to 14-fold decrease in Nrf2 mRNA level; Supplementary Figure S3) and immunocytochemical (ICC) analysis (Figure 2A). Nrf2 expression was decreased significantly in the cytosolic region of MCF-7 cells (Figure 2A). The knockdown also significantly downregulated the Nrf2 level in nuclear compartments compared to that in scrambled siRNA-transfected cell controls. Microphotographs (40× magnification) indicate a decrease in Nrf2 in both the cytosol and nucleus (Figure 2A). These results are supported by the data obtained using RT-PCR to measure the expression of Nrf2 mRNA (Supplementary Figure S3) and western blot analysis of whole cell lysates to measure Nrf2 protein expression. Our data indicated a decrease in Nrf2 RNA as well as protein in siRNA-transfected MCF-7, MDA-MB-231, and MDA-MB-468 cells compared to controls (Supplementary Figures S3 and S4). The activity of NQO1 (a direct target of Nrf2) [69] was assessed in cell lysates to further confirm successful knockdown of Nrf2. NQO1 activity was found to be significantly decreased in MDA-MB-468 and MCF-7 cells (Figure 2B).

SiRNA-transfected cells viability was tested using MTT assay at 24 h (no effects observed; data not shown), 48 h, and 72 h after transfection. Nrf2-targeting siRNAs induced a significant reduction in cell viability compared to scrambled siRNA-transfected MDA-MB-468 cells (Figure 2C). The viability was also significantly decreased in Nrf2-silenced MCF-7 cells (Figure 2C). A non-significant decrease (>10%) in cell viability was observed in Nrf2-silenced MDA-MB-231 cells (Supplementary Figure S5).

**Figure 3:**
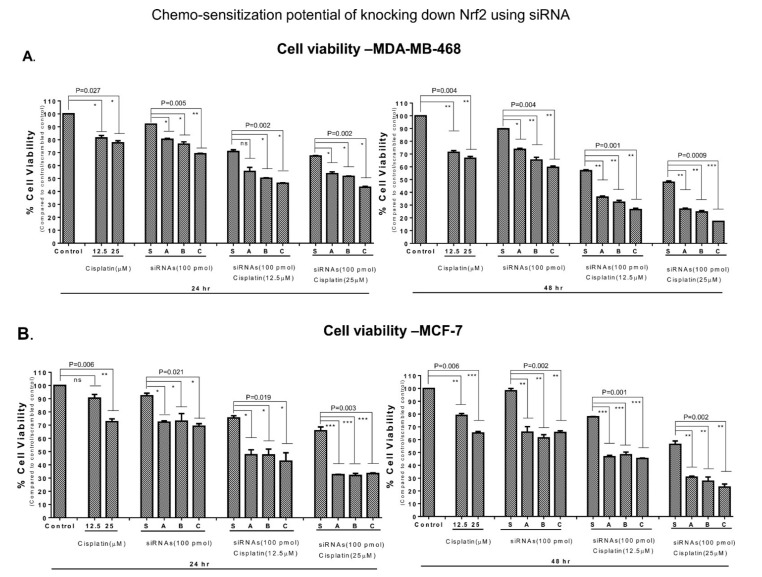
Nrf2 downregulation sensitized BC cells to chemotherapy drug cisplatin: (**A**) Nrf2 downregulation resulted in inhibition of MDA-MB-468 cells viability. Significant decrease in cell viability was observed for combined Nrf2 knockdown (siNrf2-A, siNrf2-B, and siNrf2-C) and cisplatin treatment (24 h treatment; *p* value = 0.002; 48 h of treatment; *p* value = 0.0009). (**B**) Similar effects were observed in MCF-7 cells (24 h treatment; *p* value = 0.003; 48 h of treatment; *p* value = 0.002). One-way ANOVA test was used for the data analysis (* *p* < 0.05, ** *p* < 0.01, *** *p* < 0.005). ns refers to non-significant.

Furthermore, we questioned whether Nrf2 knockdown sensitizes tumor cells to the chemotherapeutic agent cisplatin. MCF-7, MDA-MB-231, and MDA-MB-468 cells were transfected with 100 pmol of Nrf2 silencing siRNA or control scrambled RNA. Following this, untransfected and transfected cells were exposed to cisplatin (12.5 and 25 µM) for 24 and 48 h (Figure 3 and Supplementary Figure S5B). A significant decrease in cell viability was detected in cells with combined downregulation of Nrf2 and cisplatin treatment (Figure 3) compared to groups with siRNA or cisplatin alone. The viability of untransfected MDA-MB-468 cells was reduced by 28% and 33% in the presence of 12.5 and 25 µM of cisplatin, respectively (Figure 3A). This percentage was increased in cells with silenced Nrf2 that were treated with 25 µM cisplatin (82% inhibition). Similar results were observed in MCF-7 (Figure 3B) and MDA-MB-231 cells (Supplementary Figure S5B). These data indicate the downregulation of Nrf2 using siRNA sensitized cells to cisplatin. Surprisingly, decreased cell viability was also observed in some scrambled siRNA-transfected cells treated with 12.5 and 25.0 µM of cisplatin compared to cells treated with cisplatin or scrambled siRNA alone (Figure 3A). The data suggest that there is cell damage associated with the transfection of cells with scrambled siRNA loaded RNAiMAX particles.

### 3.3. Pharmacological Inhibition of Nrf2 Using Brusatol Blocked BC Cell Growth and Migration In Vitro

**Figure 4:**
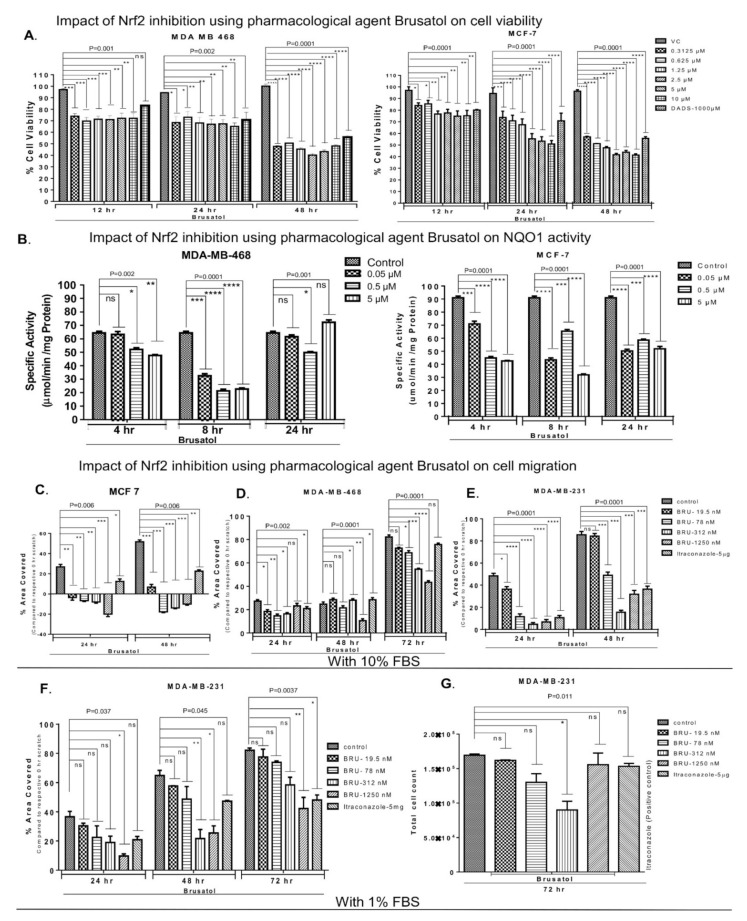
Pharmacological inhibition of Nrf2 using brusatol retarded BC cells proliferation and NQO1 activity in vitro: (**A**) cell viability was measured using SRB assay. Brusatol (concentration range from 0.3125 to 10 µM) was used as chemical inhibitor of Nrf2 [37,70]. MDA-MB-468 and MCF-7 cells were exposed to increasing concentrations of brusatol for 12, 24, and 48 h. The growth inhibition was time-dependent (*p* value = 0.0001; *p* value = 0.0001). Brusatol-induced growth inhibitory effects were compared with those in vehicle treated cells. DADS (1 mM) was used as positive control (**B**) NQO1 activity was assessed in MDA-MB-468 and MCF-7 cells treated with vehicle control or different concentrations of brusatol (0.05–5 µM). (**C**–**G**) Nrf2 inhibition by brusatol retards BC cell migration: Cells migration was assessed using a scratch assay. All cell lines were treated with non-toxic concentrations of brusatol (19.5 nM to 1250 nM) or vehicle control. (**C**–**E**) MCF-7, MDA-MB-468, and MDA-MB-231 cells were grown in media supplemented with 10% FBS. The inhibitory impact of brusatol treatment was the strongest in MCF-7 cells. (**F**) MDA-MB-231 were grown in 1% FBS. Brusatol treatment significantly decreased the percentage area covered by migrating cells. Itraconazole (5.0 µg/mL) was used as a positive control for inhibiting cell migration [61]. (**G**) Cell count was found similar in vehicle- and brusatol-treated wells. One-way ANOVA test was used for the data analysis (* *p* < 0.05, ** *p* < 0.01, *** *p* < 0.005, **** *p* < 0.001). ns refers to non-significant.

The inhibitor of Nrf2 signaling brusatol [70] activates degradation of Nrf2 in a Keap-1 independent manner [29]. MDA-MB-468, MCF-7, and MDA-MB-231 cells were treated with increasing concentrations of brusatol (from 19.5 nM to 10 µM) for 12, 24, and 48 h (Figure 4A and Supplementary Figure S5C). The effect of two low/non-toxic concentrations (19.5 and 78 nM) of brusatol did not demonstrate any significant effect on cell viability (data not shown). Brusatol provoked a significant decrease in BC cell viability; however, this effect was not dose- or time-dependent (Figure 4A and Supplementary Figure S5C). The highest cytotoxic effects of brusatol were observed at 24 and 48 h of treatment (Figure 4A).

Effects of various doses of brusatol (0.05 to 5 µM) on NQO1 activity were tested in MDA-MB-468, MCF-7, and MDA-MB-231 cell lysates at 4, 8, and 24 h of treatment (Figure 4B and Supplementary Figure S5D). Data analysis revealed dose- and time-dependent decreases in NQO1 activity at 4 and 8 h, but not at 24 h of brusatol treatment in all cell lines (Figure 4B and Supplementary Figure S5D). No further decreases in NQO1 activity were observed in MDA-MB-468 and MDA-MB-231 cells treated with higher concentrations of brusatol after 24 h treatment (Figure 4B and Supplementary Figure S5D). These observations indicate potential saturation of Nrf2 inhibition by high brusatol doses (5 μM), which warrants further investigation. Thus, brusatol-induced downregulation of NQO1 activity preceded inhibition of BC cell growth.

Next, the ability of brusatol to inhibit cancer cell migration was tested using a scratch assay [71]. Brusatol strongly inhibited MCF-7 cell migration during 48 h of incubation (Figure 4C). A significant dose-dependent decrease in percentage of area covered by migrating cells was observed for all BC cell lines (Figure 4C–E). Major changes in MDA-MB-468 cell migration were recorded 72 h after introduction of the scratch (Figure 4D). Cell migration of brusatol-treated MDA-MB-231 differed from the control at 24 h (Figure 4E). To exclude the role of cell proliferation in gap closure, MDA-MB-231 cells were also grown in 1% FBS medium as detailed previously [62,63]. Cell migration rate was reduced under low serum conditions in our experiments (Figure 4F) and reported previously [72]. Brusatol (1250 nM) significantly reduced the migration of cells in a dose-dependent manner in low-serum conditions compared to vehicle-treated controls (Figure 4F). Cell count was found to be similar in vehicle- and brusatol-treated wells (Figure 4G). Cell proliferation in closing scratch wounds was not observed (data not shown) in reduced serum concentration (1% FBS). Moreover, significantly decreased migration was observed along with a reduction in cell number in MDA-MB-231 treated with 312 nM of brusatol (Figure 4G), suggesting that this dose of brusatol also activated apoptosis. Itraconazole (5 µg/mL), an inhibitor of cell invasion and migration, served as a positive control [61]. Itraconazole treatment resulted in 60–80% migration inhibition in MDA-MB-231 and MCF-7 cell lines (Figure 4C,E). Representative microphotographs are shown in Supplementary Figure S6A–D.

### 3.4. Genetic and Pharmacological Inhibition of Nrf2 Induced Accumulation of Cells in Sub-G0-G1 and G2/M Phases of Cell Cycle

**Figure 5:**
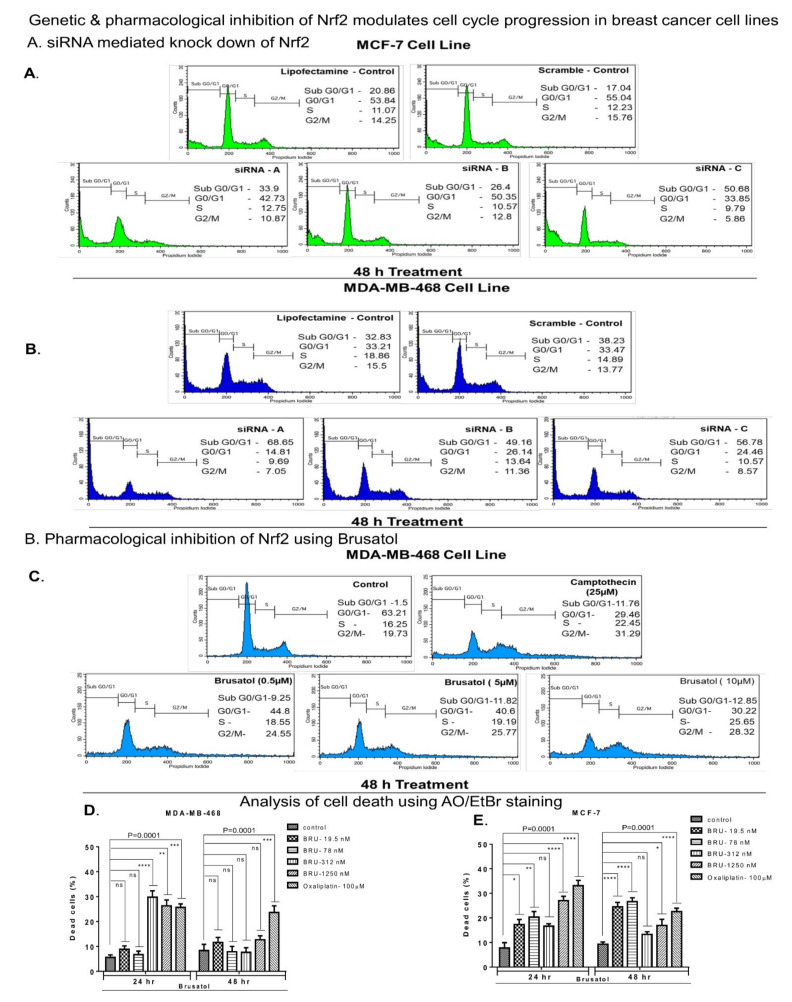
Inhibition of Nrf2 using siRNA and brusatol induced cell cycle arrest and promoted cell death in vitro: (**A**,**B**) cell cycle progression was measured using PI staining followed by FACS analysis of the stained cells. A significant increase in sub-G0-G1 cell population was detected in MCF-7 (**A**) and MDA-MB-468 (**B**) cells transfected with siRNAs targeting Nrf2. (**C**–**E**) Cell cycle using PI staining followed by analysis of stained cells using FACS (**C**) and apoptosis (**D**,**E**) were measured using AO/EB dual staining. Treatment with brusatol (0.5, 5.0, and 10.0 µM) arrested MDA-MB-468 cells (**C**) in G2/M phase and increased sub-G0-G1 cell population. A significant increase in the number of dead cells was observed in brusatol treated (19.5 nM to 1250 nM) MDA-MB-468 cells (**D**) *p* value = 0.0001) and MCF 7 cells (**E**) *p* value = 0.0001). One-way ANOVA test was used for the data analysis (* *p* < 0.05, ** *p* < 0.01, *** *p* < 0.005, **** *p* < 0.001). ns refers to non-significant.

To elucidate the mechanism of Nrf2-related effects in BC, MCF-7, and MDA-MB-468 cell lines were subjected to cell cycle analysis. To downregulate Nrf2, MCF-7, and MDA-MB-468 cells were transfected with 100 pmol of three different siRNAs (siNrf2-A, siNrf2-B, and siNrf2-C). A significant increase in sub-G0-G1 cell population was observed, along with a decrease in cell populations at other cell cycle stages (G0/G1, S, and G2/M). The effect could be associated with the elevated cell death observed in Nrf2 siRNA-transfected cells compared to control lipofectamine RNAiMAX reagent or scrambled siRNA-transfected cells (Figure 5). MCF-7 cells exposed to lipofectamine RNAiMAX reagent or transfected with scrambled siRNA had 20.86% and 17.04% of sub-G0-G1 cells, respectively (Figure 5A). The sub-G0-G1 percentage of cells increased to 33.9%, 26.4%, and 50.68% with siNrf2-A, siNrf2-B, and siNrf2-C, respectively. MDA-MB-468 cells exposed to lipofectamine RNAiMAX reagent had 32.83% of cells in the sub-G0-G1 stage, while siScrambled RNA had 38.23%. The percentage of sub-G0-G1 cells increased to 68.65%, 49.16%, and 56.78% as a result of transfecting the cells with siNrf2-A, B, and C, respectively (Figure 5B).

Cell cycle analysis was also performed using staining with propidium iodide (PI) and FACS in MDA-MB-468 cells exposed to or not exposed to Nrf2 inhibitor Brusatol (Figure 5C). A significant increase in G2/M phase cell population was detected in cells treated with 0.5, 5.0, and 10.0 µM of brusatol after 48 h of treatment (Figure 5C). A significantly increased sub-G0-G1 population was also registered in brusatol-treated cells.

### 3.5. Nrf2 Inhibitor Brusatol Stimulates BC Cell Death

The pro-apoptotic effects of brusatol were assessed using acridine orange (AO) and ethidium bromide (EB) dual staining procedure (Figure 5D,E and Supplementary Figure S7). Healthy live cells appear green due to the uptake of membrane permeable AO, while the pro-apoptotic cells are red due to the formation of broken DNA complexes with EB [73]. We found an increased percentage of dead MDA-MB-468 and MCF-7 cells treated with increasing concentrations of brusatol (19.5 to 1250 nM). The increased percentage of dead cells was not dose-dependent in either cell line (Figure 5D,E and Supplementary Figure S7A,B). Treatment with 100 µM oxaliplatin (the positive control) induced cell death in ~26% of MDA-MB-468 cells at 24 h and 48 h. However, in MCF-7, 100 µM oxaliplatin treatment induced cell death in ~33% and 23% of cells at 24 and 48 h of exposure, respectively (Figure 5E). Similar results were observed in MDA-MB-231 cells (Supplementary Figure S7C,D).

To assess the level of apoptosis in siRNA-transfected BC cells, MDA-MB-468, MCF-7, and MDA-MB-231 were transfected with 100 pmol of siRNA. Percentages of live (green), apoptotic (yellow), and dead (red) cells were counted. Analysis of the data showed a significant increase in apoptosis in MDA-MB-468 cells with downregulated Nrf2. The highest level of apoptosis was detected 72 h after transfection (Supplementary Figure S8A). An increased level of apoptosis was also observed in MCF-7 cells transfected with siNrf2-A and siNrf2-C (Supplementary Figure S8B). Downregulation of Nrf2 did not influence apoptosis in the MDA-MB-231 cell line (Supplementary Figure S8C).

### 3.6. Intraperitoneal Administration of Brusatol Inhibited EAC Solid Tumors Development in Mice In Vivo

**Figure 6:**
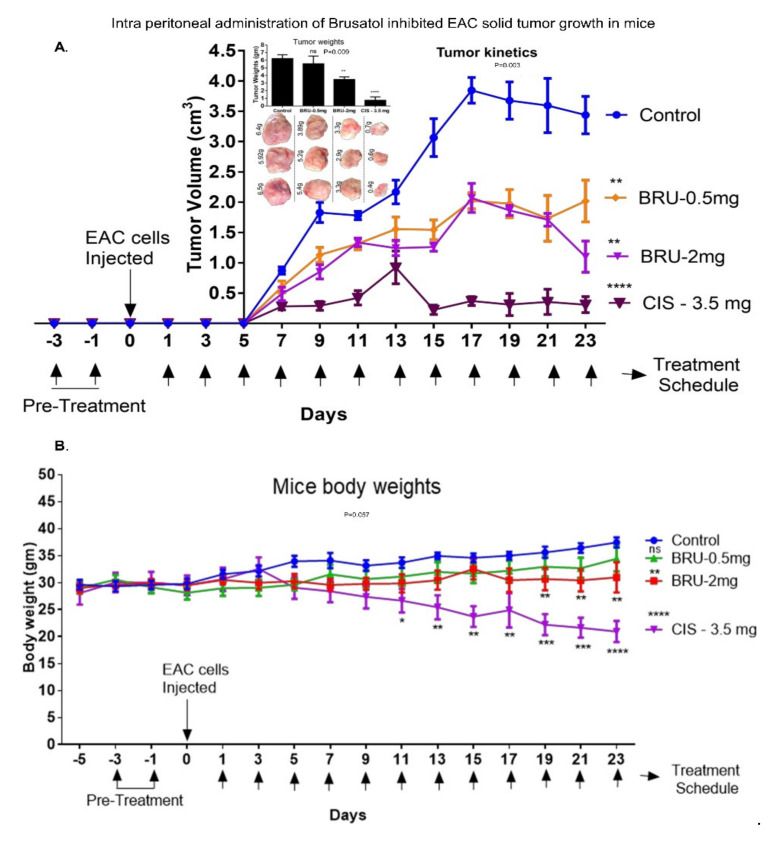
Intraperitoneal administration of Brusatol retarded EAC solid tumor growth in vivo. Swiss albino mice were engrafted with Nrf2 expressing EAC cells and treated with two different doses of brusatol (0.5 mg/kg and 2.0 mg/kg). (**A**) Brusatol (2.0 mg/kg) treatment decreased tumor growth significantly (*p* value = 0.003; at day 23). Cisplatin (3.5 mg/kg) reduced tumor volume and tumor weight effectively. (**B**) Brusatol (0.5 mg/kg) administration did not induce significant changes in body weight (*p* value = 0.05), although application of higher brusatol dose (2 mg/kg) resulted in decreased body mass after 17-day treatment. Cisplatin induced significant decreases in body mass after 12th day treatment (*p* value = 0.01). One-way ANOVA test was used for the data analysis (* *p* < 0.05, ** *p* < 0.01, *** *p* < 0.005, **** *p* < 0.001). ns refers to non-significant.

We detected high level of Nrf2 expression in EAC cells using qRT-PCR (Supplementary Figure S9A,B). EAC cells express higher Nrf2 levels than that in A549 lung carcinoma cells (Supplementary Figure S9A,B). Therefore, we used EAC cells in tumor engraftment model in vivo. EAC-engrafted mice were administered brusatol (0.5 mg/kg or 2.0 mg/kg every other day) and tumor volume was recorded (Figure 6 and Supplementary Figure S10A). Brusatol provoked a significant 40–70% reduction in tumor volume (Figure 6A), but not in animal body mass until day 17 (Figure 6B, BRU-2 mg only). Cisplatin (positive control) strongly reduced the tumor volume, although a significant reduction in body mass was also observed (day 13; Figure 6A,B). Low dose brusatol did not stimulate a significant reduction in the extracted tumor mass at day 23 compared to vehicle control group. However, higher dose of brusatol resulted in a significant reduction of tumor mass (Figure 6A and Supplementary Figure S10B). Cisplatin (3.5 mg/kg) reduced the tumor mass by 88% at day 23 (Figure 6A).

### 3.7. Brusatol Treatment Reduced Nrf2 Activity/Expression and the Activity of Nrf2-Target Gene NQO1 in Tumors In Vivo

**Figure 7:**
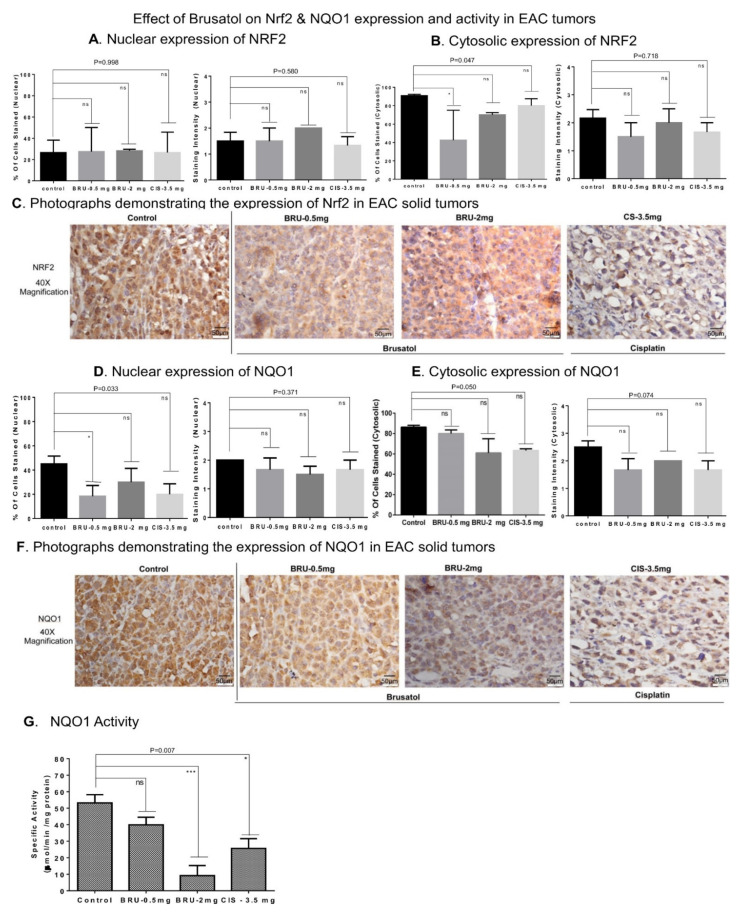
Effects of brusatol and cisplatin on Nrf2 and NQO1 expression and activity in EAC tumors ex vivo. (**A**) IHC staining analysis of Nrf2 expression and localization in engrafted EAC tumor tissues. Brusatol administration did not change the nuclear expression of Nrf2 (a; *p* value = 0.998; *p* value = 0.580). (**B**) Brusatol (0.5 mg/kg) induced a significant decrease in the number of cells with cytosolic Nrf2 expression (left panel; *p* value = 0.047). (**C**) Representative IHC microphotographs of Nrf2 stained tumor tissues are shown (Scale: 50 µm). (**D**) Brusatol (0.5 mg/kg) induced a significant decrease in number of cells expressing nuclear NQO1 (left panel; *p* value = 0.033). (**E**) The cytosolic expression of NQO1 was assessed in EAC tumors treated with brusatol and cisplatin (*p* value = 0.050 vs. control). No significant changes were observed in NQO1 staining intensity. (**F**) Representative IHC microphotographs of NQO1 stained tumor tissues are shown (Scale: 50 µm). (**G**) Brusatol (2 mg/kg) stimulated a significant decrease in NQO1 activity in the tumor lysates (*p* value = 0.007). The positive control cisplatin also reduced NQO1 activity. One-way ANOVA test was used for the data analysis ( * *p* < 0.05, *** *p* < 0.005). ns refers to non-significant.

IHC was used to determine Nrf2 and its target gene expressions in tumor-engrafted and brusatol-treated mice. No significant changes in the nuclear Nrf2 expression were detected, although the cytosolic Nrf2 expression was significantly decreased in brusatol (0.5 mg/kg)-treated animals (Figure 7A–C). Nuclear NQO1 was significantly decreased in lower dose brusatol-treated (0.5 mg/kg) tumors, compared to cisplatin (3.5 mg/kg; a nonsignificant decrease) treated animals (Figure 7D–F). Alternatively, higher brusatol doses and cisplatin stimulated significant decreases in cytosolic NQO1 (Figure 7E). No changes in the intensity of staining were observed for all treatments. To confirm the observed effects, NQO1 activity was also determined using colorimetric method [44,69]. Administration of brusatol (2.0 mg/kg) reduced NQO1 activity by 5-fold. Interestingly, a 2.5-fold decrease in NQO1 activity was also observed in tumors harvested from cisplatin-treated animals (Figure 7G).

### 3.8. Brusatol-Induced Tumor Growth Inhibition Is Mediated by Decreased Expression of Ki67, CD31, and Enhanced Lymphocyte Invasion

**Figure 8:**
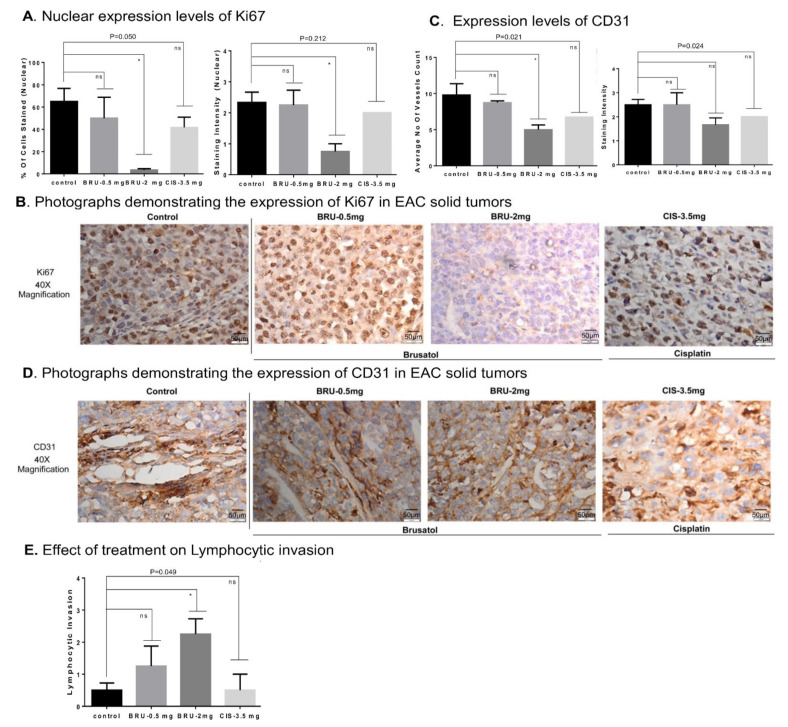
Brusatol reduced CD31 and Ki67 expression in grafted EAC tumor tissues and promoted lymphocytic invasion in vivo. (**A**,**B**) Ki67 staining/IHC was used to assess proliferation of grafted EAC [75]. Slides were scored and percentage of Ki67 positive cells was calculated per image. Administration of 2.0 mg/kg brusatol reduced nuclear expression of Ki67 (*p* value = 0.050; *p* value = 0.212). (**B**) Representative IHC microphotographs of Ki67 stained tumor tissues are shown (Scale: 50 µm). (**C**,**D**) Tumor angiogenesis was assessed using CD31 staining/IHC. Brusatol treatment reduced level of angiogenesis in vivo (*p* value = 0.021; *p* value = 0.024). (**E**) Brusatol promoted lymphocytic invasion (*p* value = 0.049). Cisplatin had minimal effects on expression of Ki67 and CDE31 (a and c). (**D**) Representative IHC microphotographs of CD31 stained EAC tumor tissues are shown (Scale: 50 µm). One-way ANOVA test was used for the data analysis (* *p* < 0.05). ns refers to non-significant.

To assess the cell division rate, the number of proliferating cells was quantified using Ki67 staining [74]. Analysis of Ki67 staining intensity confirmed the nuclear Ki67 expression pattern (Figure 8A,B). We detected a significant decrease in the percentage of Ki67-positive tumor cells from mice in 2.0 mg/kg brusatol-treated animals (Figure 8A). In cisplatin-treated animals, a non-significant decrease in percentage of Ki67 positive cells was observed (62% in control vs. 40% in cisplatin group; Figure 8A).

The blood vessel number, indicated by CD31 + cells, was significantly decreased in 2.0 mg/kg brusatol-treated animals compared to controls (Figure 8C). Staining intensity assessment showed no significant reductions in CD31 expression (Figure 8C,D). Cisplatin (3.5 mg/kg) treatment did not induce significant decreases in the number of CD31 + cells (Figure 8C,D). Low dose brusatol (0.5 mg/kg) showed no significant decreases in CD31 + vessel density (Figure 8C). However, the lymphocytic invasion was elevated in brusatol-treated tumors (Figure 8E). The lymphocyte invasion score was higher in tumors exposed to 2.0 mg/kg of brusatol compared to 0.5 mg/kg brusatol-treated animals (Figure 8E). Brusatol-induced stimulation of lymphocyte migration indicates an activation of anti-tumor immune responses in Nrf2-inhibited tumors.

## 4. Discussion

Our data support a growing body of evidence indicating that Nrf2 is a key tumor-regulating effector. We detected elevated nuclear Nrf2 expression and activity in advanced grade II and III BCs (Figure 1), confirming cancer-promoting functions of Nrf2. Other recent studies have reported an elevated Nrf2 expression in BC tissues and cells, although the correlation between Nrf2 expression, BC treatment-related markers (ER, PR, and HER2), and BC cell sensitivity to apoptosis were not addressed comprehensively [23,31,76]. In this study we determined that Nrf2 expression levels are elevated in triple negative BCs (Supplementary Table S1), although the difference in Nrf2 expression was not found to be significant (normal breast tissues (n = 3) vs. BCs (n = 46)). Furthermore, we detected induction of apoptosis in BC cells stimulated with pharmacological Nrf2 inhibitor brusatol (Figure 5). SiRNAs-induced inhibition of Nrf2 resulted in increased sensitivity to cisplatin treatment (Figure 3). Nrf2 knockdown was marked by inhibition of its downstream effector NQO1 (the oxidative stress regulator) [77]. Nrf2–NQO1 signaling pair was linked to the detoxification of quinone substrates, highly reactive compounds associated with both apoptosis and carcinogenesis [78].

Previous investigations demonstrated that targeted inhibition of Nrf2 blocked migration of cancer cells and sensitized them to chemotherapy [79,80]. For instance, Nrf2 knockdown sensitized A549 lung cancer cells to cisplatin, doxorubicin, and etoposide [81]. Pharmacological inhibition of Nrf2 using trigonelline sensitized pancreatic cells to apoptosis [82]. Synergistic anti-cancer effects of brusatol and trastuzumab (HER2 targeting agent) were reported in HER2 positive BC and associated with induction of reactive oxygen species (ROS) production and apoptosis [83]. Furthermore, cooperative effects of brusatol and other anticancer agents, including gemcitabine (in pancreatic carcinomas) [84], cytarabine (Ara-C) (in acute myeloid leukemia) [85], luteolin (in gastric cancers) [86], and paclitaxel (in BCs) [87,88], were observed. Other studies supported clinical applications of Nrf2 inhibition by brusatol in different cancers [30,35,89]. The disruption of Nrf2 nuclear export prevented the nuclear accumulation of this protein and sensitized A549 cells to cisplatin and carboplatin [80]. The expression of Nrf2 was estimated using IHC that indicated nuclear localization of Nrf2 in 44% of BCs [76]. Nrf2 was suggested to serve as an independent adverse prognostic factor for both recurrence and disease-free survival [76,90]. IHC data from this study has also shown elevated nuclear accumulation of Nrf2 in grade II and III BCs compared to normal breast cells highlighting the potential of nuclear Nrf2 to serve as a biomarker protein.

Enhanced Nrf2 expression has been associated with activation of Nrf2 downstream signaling effectors [58]. Under oxidative stress conditions, Nrf2 was shown to translocate into the nucleus and trigger the transcription of antioxidant response proteins, including NQO1, SOD, and catalase [91]. Accordingly, we found that brusatol-induced Nrf2 inhibition resulted in reduced NQO1 activity (Figure 4). Furthermore, a link between inhibition of NQO1 activity and activation of apoptosis was observed previously [92]. Our data indicate that inhibition of Nrf2/NQO1 signaling results in cell cycle arrest and decreased cell viability. The inhibited cell proliferation may be associated with cell cycle arrest [93]. Inhibition of Nrf2 using siRNA is also known to induce apoptosis [70]. Genetic disruption of Nrf2 expression in alveolar epithelium induced cell cycle arrest in the G2/M phase [94]. Interestingly, we also detected G2/M arrest in MDA-MB-468 cells treated with brusatol (Figure 5). However, no G2/M arrest was observed when Nrf2 was downregulated by siRNA. This discrepancy may be caused by the siRNA-mediated downregulation of Nrf2, which coincided with the increased size of sub-G0-G1 population that could compensate for the earlier cell deaths. Similar effects were observed previously [95], as G2/M arrest is one of the early events before a cell undergoes apoptosis [96]. However, the mechanism of the direct activation of apoptosis/cell death by brusatol-dependent inhibition of Nrf2 and downregulation of NQO1 activity in BC cells remains to determine.

We are the first to report that EAC cells express high levels of Nrf2 and respond to brusatol treatment in Swiss albino mice. Brusatol treatment in vivo significantly reduced engrafted EAC tumor volume, while inducing much smaller changes in body mass, compared to cisplatin, which significantly reduced body weight in our experiments (Figure 6). Our data indicate less pronounced systemic effects of brusatol compared to cisplatin-induced effects, although systemic toxicity of brusatol requires further investigation. The previous in vivo study used A549 lung cancer tumors with genetically lowered Nrf2 expression, which sensitized cancer cells to cisplatin/carboplatin pro-apoptotic effects in nude mice [37]. Using CRISPR-Cas9 technology, another study demonstrated that xenografted Nrf2-null lung tumors develop slower and exhibit increased sensitivity to chemotherapy [80]. Pharmacological inhibition of Nrf2 with brusatol has been shown to reduce colon tumor growth in mice [30].

Nrf2 scavenges reactive oxygen species, promotes cell survival, inhibits apoptosis, and increases cell proliferation and migration [97,98]. Additionally, Nrf2 increases anti-cancer drug resistance, especially under hypoxic conditions [19]. Hypoxic conditions were associated with the activation of Nrf2, which facilitated the development of cisplatin resistance in MCF-7 cells [99]. Initiated by downregulation and inhibition of Nrf2, chemosensitization to cisplatin was also observed in the present study. The effect of anti-cancer drug chemosensitization can be mediated by the enhanced level of ROS, one of the most powerful pro-apoptotic stimulators [100]. Suggestively, the level of ROS maybe increased when the level of Nrf2 (oxidative stress repressor) is low or downregulated in the presence of siRNAs. Supporting this hypothesis, constitutive Nrf2 activation was shown to defend malignant cells against oxidative stress [101]. However, the role of ROS requires further investigation in brusatol-treated BC cells.

Nrf2 signaling facilitates BC cell migration [21] and metastasis [102]. It has been demonstrated that overexpression of Nrf2 in MCF-7 and MDA-MB-231 resulted in elevated G6PD, HIF1α, Notch1, and HES1 signaling [103]. The same study detected enhanced cell migration and proliferation in Nrf2 overexpressing BC cells. Tumor progression also relies on angiogenesis and decreased anti-tumor lymphocyte invasion (non-aggressive tumor microenvironment) [104,105]. In the present study, a significant decrease in tumor cell migration and proliferation was observed in cells exposed to the Nrf2 inhibitor brusatol (Figure 4). Furthermore, we observed a reduction in blood vessel/CD31+ staining in brusatol-exposed tumors, while the lymphocytic invasion was elevated (Figure 8). The lymphocyte invasion score was higher in tumors exposed to 2.0 mg/kg of brusatol, suggesting that Nrf2 inhibition stimulates lymphocyte migration and potentially activates anti-tumor immunity (Figure 8). Activated lymphocyte migration may be a sign of increased cell aggressiveness in the tumor microenvironment. However, this effect requires further investigation. Co-staining with specific tumor-infiltrating lymphocyte marker and anti-Nrf2 antibodies maybe used in future studies to address the role of Nrf2 expression in activation of lymphocyte trafficking.

In conclusion, our data indicate that there is a tendency for increased Nrf2 levels in a set of BC tissues. This observation suggests a need to test Nrf2 expression in a larger set of normal and BC tissues to confirm potential benefit of targeted Nrf2 inhibition. The observed anti-migratory and pro-apoptotic effects of brusatol support this hypothesis. Larger clinical investigations are warranted to confirm percentage of BC patients who will benefit from Nrf2-targeting treatment. The development and testing of non-toxic and more efficient Nrf2 inhibitors is needed. The benefits of combined treatment (brusatol and other anticancer agents) should be assessed in advanced BCs. This study demonstrated that EAC-based in vivo model could be successfully used for differential testing of Nrf2 inhibitors.

## 5. Conclusions

The results of our study demonstrate that the higher-grade BC tissues have a tendency to express enhanced levels of Nrf2. Nuclear Nrf2 localization was increased in advanced BCs. Targeted inhibition of Nrf2 using siRNA or the pharmacological agent brusatol sensitized BC cells to cisplatin, inhibited BC cell migration and division, decreased xenografted tumor growth, and stimulated lymphocyte migration towards tumor tissues in mice in vivo. Our data support biomarker characteristics of Nrf2 in advanced BCs. Nrf2 inhibition triggers cell cycle arrest and sensitizes tumor cells to pro-apoptotic and growth-inhibiting effects of chemotherapeutic agents.

## Figures and Tables

**Table 1:** Used primer sequences *.

Sno:	Gene Name	Forward Primer	Reverse Primer	Product Size (bp)	Ref.
Primer sequences used for Homo sapiens					
1	*NRF2*	TTCAGCAGCATCCTCTC CACAG	GCATGCTGTTGCTGATACTGG	139	[48]
2	*NQO1*	TGCAGCGGCTTTGAAG AAGAAAGG	TCGGCAGGATACTGAAAGTTCGCA	251	[49]
3	*β-Actin*	TGGATCAGCAAGCAGG AGTATG	GCATTTGCGGTGGACGAT	57	[50]
4	*GAPDH*	CGACCACTTTGTCAAGC TCA	AGGGGAGATTCAGTGTGGTG	307	[51]
Primer sequences for mouse EAC cells					
5	*NRF2*	TTCTTTCAGCAGCATCC TCTCCAC	ACAGCCTTCAATAGTC CCGTCCAG	199	[52]
6	*NQO1*	TATCCTTCCGAGTCATC TCAGC	TCTGCAGCTTCCA GCTTCTTG	86	[53]
7	*GAPDH*	AGAGAGGGAGGAGGG GAATG	AACAGGGAGGAGCA GAGAGCAC	200	[54]

* qRT-PCR experiment was performed using these primer sequences.

## Data Availability

Exclude this statement as this study did not report any data.

## Supplementary Materials

The following are available online at https://www.mdpi.com/article/10.3390/biomedicines9091119/s1. Supplementary Figure S1: Schematic representation of the procedure for preparing tissue array. Supplementary Figure S2: Flow chart depicting the procedure for preparing cell blocks. Supplementary Figure S3: siRNA-mediated knockdown of Nrf2 reduced its expression at mRNA level in breast cancer cell lines. Supplementary Figure S4: Targeting Nrf2 using siRNA reduced the expression of Nrf2 at protein level. Supplementary Figure S5: Targeting Nrf2 using siRNAs reduced the viability of MDA-MB-231 breast cancer cells in vitro. Supplementary Figure S6: Pharmacological inhibition of Nrf2 using brusatol retarded cell migration in breast cancer cells. Supplementary Figure S7: Treatment with brusatol induced death in breast cancer cells. Supplementary Figure S8: Targeted inhibition of Nrf2 using siRNA induced cell death in breast cancer cells. Supplementary Figure S9: EAC cells expressed elevated Nrf2 compared to positive control A549 cell line. Supplementary Figure S10: Schematic representation of in vivo study protocol and the photographs of mice. Supplementary Table S1: Immunohistochemical staining scores of normal, grade-II and grade-III breast cancer tissues. Table S2: Nucleotide sequence of siRNAs used in this study. Supplementary Table S3: The clinical features of the grade-II and grade-III tumors and keys to master chart.

## References

[1] DeSantis C.E., Ma J., Gaudet M.M., Newman L.A., Miller K.D., Goding Sauer A., Jemal A., Siegel R.L. (2019). Breast cancer statistics, 2019. CA Cancer J. Clin..

[2] Bayraktar S., Batoo S., Okuno S., Gluck S. (2019). Immunotherapy in breast cancer. J. Carcinog..

[3] Masoud V., Pages G. (2017). Targeted therapies in breast cancer: New challenges to fight against resistance. World J. Clin. Oncol..

[4] Yoo B., Fuchs B.C., Medarova Z. (2018). New Directions in the Study and Treatment of Metastatic Cancer. Front. Oncol..

[5] Koury J., Lucero M., Cato C., Chang L., Geiger J., Henry D., Hernandez J., Hung F., Kaur P., Teskey G. (2018). Immunotherapies: Exploiting the Immune System for Cancer Treatment. J. Immunol. Res..

[6] Cai F., Luis M.A.F., Lin X., Wang M., Cai L., Cen C., Biskup E. (2019). Anthracycline-induced cardiotoxicity in the chemotherapy treatment of breast cancer: Preventive strategies and treatment. Mol. Clin. Oncol..

[7] Saloustros E., Mavroudis D., Georgoulias V. (2008). Paclitaxel and docetaxel in the treatment of breast cancer. Expert Opin. Pharmacother..

[8] Wahba H.A., El-Hadaad H.A. (2015). Current approaches in treatment of triple-negative breast cancer. Cancer Biol. Med..

[9] Hernandez-Aya L.F., Ma C.X. (2016). Chemotherapy principles of managing stage IV breast cancer in the United States. Chin. Clin. Oncol..

[10] Tong C.W.S., Wu M., Cho W.C.S., To K.K.W. (2018). Recent Advances in the Treatment of Breast Cancer. Front. Oncol..

[11] Taherkhani M., Mahjoub S., Moslemi D., Karkhah A. (2017). Three cycles of AC chemotherapy regimen increased oxidative stress in breast cancer patients: A clinical hint. Caspian J. Intern. Med..

[12] Cocconi G., Bisagni G., Bella M., Acito L., Anastasi P., Carpi A., Di Costanzo F., Frassoldati A., Mosconi A., Borrini A. (1999). Comparison of CMF (cyclophosphamide, methotrexate, and 5-fluorouracil) with a rotational crossing and a sequential intensification regimen in advanced breast cancer: A prospective randomized study. Am. J. Clin. Oncol..

[13] Nurgali K., Jagoe R.T., Abalo R. (2018). Editorial: Adverse Effects of Cancer Chemotherapy: Anything New to Improve Tolerance and Reduce Sequelae?. Front. Pharmacol..

[14] Fayanju O.M., Park K.U., Lucci A. (2018). Molecular Genomic Testing for Breast Cancer: Utility for Surgeons. Ann. Surg. Oncol..

[15] Green N., Al-Allak A., Fowler C. (2019). Benefits of introduction of Oncotype DX((R)) testing. Ann. R Coll. Surg. Engl..

[16] Wuerstlein R., Kates R., Gluz O., Grischke E.M., Schem C., Thill M., Hasmueller S., Kohler A., Otremba B., Griesinger F. (2019). Strong impact of MammaPrint and BluePrint on treatment decisions in luminal early breast cancer: Results of the WSG-PRIMe study. Breast Cancer Res. Treat..

[17] Saha S., Buttari B., Panieri E., Profumo E., Saso L. (2020). An Overview of Nrf2 Signaling Pathway and Its Role in Inflammation. Molecules.

[18] Hayes J.D., McMahon M., Chowdhry S., Dinkova-Kostova A.T. (2010). Cancer chemoprevention mechanisms mediated through the Keap1-Nrf2 pathway. Antioxid. Redox Signal..

[19] Jessen C., Kress J.K.C., Baluapuri A., Hufnagel A., Schmitz W., Kneitz S., Roth S., Marquardt A., Appenzeller S., Ade C.P. (2020). The transcription factor NRF2 enhances melanoma malignancy by blocking differentiation and inducing COX2 expression. Oncogene.

[20] Tonelli C., Chio I.I.C., Tuveson D.A. (2018). Transcriptional Regulation by Nrf2. Antioxid. Redox Signal..

[21] Zhang C., Wang H.J., Bao Q.C., Wang L., Guo T.K., Chen W.L., Xu L.L., Zhou H.S., Bian J.L., Yang Y.R. (2016). NRF2 promotes breast cancer cell proliferation and metastasis by increasing RhoA/ROCK pathway signal transduction. Oncotarget.

[22] De Blasio A., Di Fiore R., Pratelli G., Drago-Ferrante R., Saliba C., Baldacchino S., Grech G., Scerri C., Vento R., Tesoriere G. (2020). A loop involving NRF2, miR-29b-1-5p and AKT, regulates cell fate of MDA-MB-231 triple-negative breast cancer cells. J. Cell Physiol..

[23] Qin S., He X., Lin H., Schulte B.A., Zhao M., Tew K.D., Wang G.Y. (2021). Nrf2 inhibition sensitizes breast cancer stem cells to ionizing radiation via suppressing DNA repair. Free Radic. Biol. Med..

[24] Gorrini C., Baniasadi P.S., Harris I.S., Silvester J., Inoue S., Snow B., Joshi P.A., Wakeham A., Molyneux S.D., Martin B. (2013). BRCA1 interacts with Nrf2 to regulate antioxidant signaling and cell survival. J. Exp. Med..

[25] Ryoo I.G., Choi B.H., Ku S.K., Kwak M.K. (2018). High CD44 expression mediates p62-associated NFE2L2/NRF2 activation in breast cancer stem cell-like cells: Implications for cancer stem cell resistance. Redox. Biol..

[26] Kim J.H., Yu S., Chen J.D., Kong A.N. (2013). The nuclear cofactor RAC3/AIB1/SRC-3 enhances Nrf2 signaling by interacting with transactivation domains. Oncogene.

[27] Bellezza I., Mierla A.L., Minelli A. (2010). Nrf2 and NF-kappaB and Their Concerted Modulation in Cancer Pathogenesis and Progression. Cancers.

[28] Rushworth S.A., Zaitseva L., Murray M.Y., Shah N.M., Bowles K.M., MacEwan D.J. (2012). The high Nrf2 expression in human acute myeloid leukemia is driven by NF-kappaB and underlies its chemo-resistance. Blood.

[29] Panieri E., Saso L. (2019). Potential Applications of NRF2 Inhibitors in Cancer Therapy. Oxid. Med. Cell Longev..

[30] Evans J.P., Winiarski B.K., Sutton P.A., Jones R.P., Ressel L., Duckworth C.A., Pritchard D.M., Lin Z.X., Fretwell V.L., Tweedle E.M. (2018). The Nrf2 inhibitor brusatol is a potent antitumour agent in an orthotopic mouse model of colorectal cancer. Oncotarget.

[31] Kovacs P., Csonka T., Kovacs T., Sari Z., Ujlaki G., Sipos A., Karanyi Z., Szeocs D., Hegedus C., Uray K. (2019). Lithocholic Acid, a Metabolite of the Microbiome, Increases Oxidative Stress in Breast Cancer. Cancers.

[32] Lu K., Alcivar A.L., Ma J., Foo T.K., Zywea S., Mahdi A., Huo Y., Kensler T.W., Gatza M.L., Xia B. (2017). NRF2 Induction Supporting Breast Cancer Cell Survival Is Enabled by Oxidative Stress-Induced DPP3-KEAP1 Interaction. Cancer Res..

[33] Robledinos-Anton N., Fernandez-Gines R., Manda G., Cuadrado A. (2019). Activators and Inhibitors of NRF2: A Review of Their Potential for Clinical Development. Oxid. Med. Cell. Longev..

[34] Wang T., Dou Y., Lin G., Li Q., Nie J., Chen B., Xie J., Su Z., Zeng H., Chen J. (2021). The anti-hepatocellular carcinoma effect of Brucea javanica oil in ascitic tumor-bearing mice: The detection of brusatol and its role. Biomed. Pharmacother..

[35] Cai S.J., Liu Y., Han S., Yang C. (2019). Brusatol, an NRF2 inhibitor for future cancer therapeutic. Cell Biosci..

[36] Panieri E., Buha A., Telkoparan-Akillilar P., Cevik D., Kouretas D., Veskoukis A., Skaperda Z., Tsatsakis A., Wallace D., Suzen S. (2020). Potential Applications of NRF2 Modulators in Cancer Therapy. Antioxidants.

[37] Ren D., Villeneuve N.F., Jiang T., Wu T., Lau A., Toppin H.A., Zhang D.D. (2011). Brusatol enhances the efficacy of chemotherapy by inhibiting the Nrf2-mediated defense mechanism. Proc. Natl. Acad. Sci. USA.

[38] Preul M.C., Stratford J., Bertrand G., Feindel W. (1993). Neurosurgeon as innovator: William V. Cone (1897–1959). J. Neurosurg..

[39] Prashanth T., Avin B.R.V., Thirusangu P., Ranganatha V.L., Prabhakar B.T., Sharath Chandra J.N.N., Khanum S.A. (2019). Synthesis of coumarin analogs appended with quinoline and thiazole moiety and their apoptogenic role against murine ascitic carcinoma. Biomed. Pharmacother..

[40] Goud K.I., Dayakar S., Vijayalaxmi K., Babu S.J., Reddy P.V. (2012). Evaluation of HER-2/neu status in breast cancer specimens using immunohistochemistry (IHC) & fluorescence in-situ hybridization (FISH) assay. Indian J. Med. Res..

[41] Krishnamurthy J., Kumar P.S. (2016). Significance of prognostic indicators in infiltrating duct carcinoma breast: Scenario in developing country. Indian J. Cancer.

[42] Gowda R., Madhunapantula S.V., Kuzu O.F., Sharma A., Robertson G.P. (2014). Targeting multiple key signaling pathways in melanoma using leelamine. Mol. Cancer Ther..

[43] Madhunapantula S.V., Sharma A., Robertson G.P. (2007). PRAS40 deregulates apoptosis in malignant melanoma. Cancer Res..

[44] Prochaska H.J., Santamaria A.B. (1988). Direct measurement of NAD(P)H:quinone reductase from cells cultured in microtiter wells: A screening assay for anticarcinogenic enzyme inducers. Anal. Biochem..

[45] Livak K.J., Schmittgen T.D. (2001). Analysis of relative gene expression data using real-time quantitative PCR and the 2(-Delta Delta C(T)) Method. Methods.

[46] Chomczynski P., Sacchi N. (2006). The single-step method of RNA isolation by acid guanidinium thiocyanate-phenol-chloroform extraction: Twenty-something years on. Nat. Protoc..

[47] Aranda P.S., LaJoie D.M., Jorcyk C.L. (2012). Bleach gel: A simple agarose gel for analyzing RNA quality. Electrophoresis.

[48] Zou X., Gao J., Zheng Y., Wang X., Chen C., Cao K., Xu J., Li Y., Lu W., Liu J. (2014). Zeaxanthin induces Nrf2-mediated phase II enzymes in protection of cell death. Cell Death Dis..

[49] Seng S., Avraham H.K., Birrane G., Jiang S., Li H., Katz G., Bass C.E., Zagozdzon R., Avraham S. (2009). NRP/B mutations impair Nrf2-dependent NQO1 induction in human primary brain tumors. Oncogene.

[50] Wang R., An J., Ji F., Jiao H., Sun H., Zhou D. (2008). Hypermethylation of the Keap1 gene in human lung cancer cell lines and lung cancer tissues. Biochem. Biophys. Res. Commun..

[51] Lister A., Nedjadi T., Kitteringham N.R., Campbell F., Costello E., Lloyd B., Copple I.M., Williams S., Owen A., Neoptolemos J.P. (2011). Nrf2 is overexpressed in pancreatic cancer: Implications for cell proliferation and therapy. Mol. Cancer.

[52] Vargas M.R., Johnson D.A., Sirkis D.W., Messing A., Johnson J.A. (2008). Nrf2 activation in astrocytes protects against neurodegeneration in mouse models of familial amyotrophic lateral sclerosis. J. Neurosci..

[53] Nam S.T., Hwang J.H., Kim D.H., Park M.J., Lee I.H., Nam H.J., Kang J.K., Kim S.K., Hwang J.S., Chung H.K. (2014). Role of NADH: Quinone oxidoreductase-1 in the tight junctions of colonic epithelial cells. BMB Rep..

[54] Hendrickx A., Pierrot N., Tasiaux B., Schakman O., Kienlen-Campard P., De Smet C., Octave J.N. (2014). Epigenetic regulations of immediate early genes expression involved in memory formation by the amyloid precursor protein of Alzheimer disease. PLoS ONE.

[55] Madhunapantula S.V., Desai D., Sharma A., Huh S.J., Amin S., Robertson G.P. (2008). PBISe, a novel selenium-containing drug for the treatment of malignant melanoma. Mol. Cancer Ther..

[56] Orellana E.A., Kasinski A.L. (2016). Sulforhodamine B (SRB) Assay in Cell Culture to Investigate Cell Proliferation. Bio Protoc..

[57] Van Meerloo J., Kaspers G.J., Cloos J. (2011). Cell sensitivity assays: The MTT assay. Methods Mol. Biol.

[58] Hrgovic I., Doll M., Kleemann J., Wang X.F., Zoeller N., Pinter A., Kippenberger S., Kaufmann R., Meissner M. (2016). The histone deacetylase inhibitor trichostatin a decreases lymphangiogenesis by inducing apoptosis and cell cycle arrest via p21-dependent pathways. BMC Cancer.

[59] Liang C.C., Park A.Y., Guan J.L. (2007). In vitro scratch assay: A convenient and inexpensive method for analysis of cell migration in vitro. Nat. Protoc..

[60] Varankar S.S., Bapat S.A. (2018). Migratory Metrics of Wound Healing: A Quantification Approach for in vitro Scratch Assays. Front. Oncol..

[61] Chen K., Cheng L., Qian W., Jiang Z., Sun L., Zhao Y., Zhou Y., Zhao L., Wang P., Duan W. (2018). Itraconazole inhibits invasion and migration of pancreatic cancer cells by suppressing TGF-beta/SMAD2/3 signaling. Oncol. Rep..

[62] Jonkman J.E., Cathcart J.A., Xu F., Bartolini M.E., Amon J.E., Stevens K.M., Colarusso P. (2014). An introduction to the wound healing assay using live-cell microscopy. Cell Adh. Migr..

[63] Grada A., Otero-Vinas M., Prieto-Castrillo F., Obagi Z., Falanga V. (2017). Research Techniques Made Simple: Analysis of Collective Cell Migration Using the Wound Healing Assay. J. Investig. Dermatol..

[64] Pujani M., Jain H., Chauhan V., Agarwal C., Singh K., Singh M. (2020). Evaluation of Tumor infiltrating lymphocytes in breast carcinoma and their correlation with molecular subtypes, tumor grade and stage. Breast Dis..

[65] Thirusangu P., Vigneshwaran V., Prashanth T., Vijay Avin B.R., Malojirao V.H., Rakesh H., Khanum S.A., Mahmood R., Prabhakar B.T. (2017). BP-1T, an antiangiogenic benzophenone-thiazole pharmacophore, counteracts HIF-1 signalling through p53/MDM2-mediated HIF-1alpha proteasomal degradation. Angiogenesis.

[66] Faustino-Rocha A., Oliveira P.A., Pinho-Oliveira J., Teixeira-Guedes C., Soares-Maia R., da Costa R.G., Colaco B., Pires M.J., Colaco J., Ferreira R. (2013). Estimation of rat mammary tumor volume using caliper and ultrasonography measurements. Lab. Anim..

[67] Singh A., Misra V., Thimmulappa R.K., Lee H., Ames S., Hoque M.O., Herman J.G., Baylin S.B., Sidransky D., Gabrielson E. (2006). Dysfunctional KEAP1-NRF2 interaction in non-small-cell lung cancer. PLoS Med..

[68] Wang T., Larcher L.M., Ma L., Veedu R.N. (2018). Systematic Screening of Commonly Used Commercial Transfection Reagents towards Efficient Transfection of Single-Stranded Oligonucleotides. Molecules.

[69] Shen J., Rasmussen M., Dong Q.R., Tepel M., Scholze A. (2017). Expression of the NRF2 Target Gene NQO1 Is Enhanced in Mononuclear Cells in Human Chronic Kidney Disease. Oxid. Med. Cell Longev..

[70] Olayanju A., Copple I.M., Bryan H.K., Edge G.T., Sison R.L., Wong M.W., Lai Z.Q., Lin Z.X., Dunn K., Sanderson C.M. (2015). Brusatol provokes a rapid and transient inhibition of Nrf2 signaling and sensitizes mammalian cells to chemical toxicity-implications for therapeutic targeting of Nrf2. Free Radic. Biol. Med..

[71] Kabala-Dzik A., Rzepecka-Stojko A., Kubina R., Jastrzebska-Stojko Z., Stojko R., Wojtyczka R.D., Stojko J. (2017). Migration Rate Inhibition of Breast Cancer Cells Treated by Caffeic Acid and Caffeic Acid Phenethyl Ester: An In Vitro Comparison Study. Nutrients.

[72] Yue P.Y., Leung E.P., Mak N.K., Wong R.N. (2010). A simplified method for quantifying cell migration/wound healing in 96-well plates. J. Biomol. Screen.

[73] Atale N., Gupta S., Yadav U.C., Rani V. (2014). Cell-death assessment by fluorescent and nonfluorescent cytosolic and nuclear staining techniques. J. Microsc..

[74] Fung A.S., Jonkman J., Tannock I.F. (2012). Quantitative immunohistochemistry for evaluating the distribution of Ki67 and other biomarkers in tumor sections and use of the method to study repopulation in xenografts after treatment with paclitaxel. Neoplasia.

[75] Basilio-de-Oliveira R.P., Pannain V.L. (2015). Prognostic angiogenic markers (endoglin, VEGF, CD31) and tumor cell proliferation (Ki67) for gastrointestinal stromal tumors. World J. Gastroenterol..

[76] Onodera Y., Motohashi H., Takagi K., Miki Y., Shibahara Y., Watanabe M., Ishida T., Hirakawa H., Sasano H., Yamamoto M. (2014). NRF2 immunolocalization in human breast cancer patients as a prognostic factor. Endocr. Relat. Cancer.

[77] Favreau L.V., Pickett C.B. (1995). The rat quinone reductase antioxidant response element. Identification of the nucleotide sequence required for basal and inducible activity and detection of antioxidant response element-binding proteins in hepatoma and non-hepatoma cell lines. J. Biol. Chem..

[78] Giudice A., Barbieri A., Bimonte S., Cascella M., Cuomo A., Crispo A., D’Arena G., Galdiero M., Della Pepa M.E., Botti G. (2019). Dissecting the prevention of estrogen-dependent breast carcinogenesis through Nrf2-dependent and independent mechanisms. Oncol. Targets Ther..

[79] Singh A., Boldin-Adamsky S., Thimmulappa R.K., Rath S.K., Ashush H., Coulter J., Blackford A., Goodman S.N., Bunz F., Watson W.H. (2008). RNAi-mediated silencing of nuclear factor erythroid-2-related factor 2 gene expression in non-small cell lung cancer inhibits tumor growth and increases efficacy of chemotherapy. Cancer Res..

[80] Bialk P., Wang Y., Banas K., Kmiec E.B. (2018). Functional Gene Knockout of NRF2 Increases Chemosensitivity of Human Lung Cancer A549 Cells In Vitro and in a Xenograft Mouse Model. Mol. Ther. Oncolytics.

[81] Wang X.J., Sun Z., Villeneuve N.F., Zhang S., Zhao F., Li Y., Chen W., Yi X., Zheng W., Wondrak G.T. (2008). Nrf2 enhances resistance of cancer cells to chemotherapeutic drugs, the dark side of Nrf2. Carcinogenesis.

[82] Arlt A., Sebens S., Krebs S., Geismann C., Grossmann M., Kruse M.L., Schreiber S., Schafer H. (2013). Inhibition of the Nrf2 transcription factor by the alkaloid trigonelline renders pancreatic cancer cells more susceptible to apoptosis through decreased proteasomal gene expression and proteasome activity. Oncogene.

[83] Yang Y., Tian Z., Guo R., Ren F. (2020). Nrf2 Inhibitor, Brusatol in Combination with Trastuzumab Exerts Synergistic Antitumor Activity in HER2-Positive Cancers by Inhibiting Nrf2/HO-1 and HER2-AKT/ERK1/2 Pathways. Oxid. Med. Cell Longev..

[84] Xiang Y., Ye W., Huang C., Yu D., Chen H., Deng T., Zhang F., Lou B., Zhang J., Shi K. (2018). Brusatol Enhances the Chemotherapy Efficacy of Gemcitabine in Pancreatic Cancer via the Nrf2 Signalling Pathway. Oxid. Med. Cell Longev..

[85] Karathedath S., Rajamani B.M., Musheer Aalam S.M., Abraham A., Varatharajan S., Krishnamurthy P., Mathews V., Velayudhan S.R., Balasubramanian P. (2017). Role of NF-E2 related factor 2 (Nrf2) on chemotherapy resistance in acute myeloid leukemia (AML) and the effect of pharmacological inhibition of Nrf2. PLoS ONE.

[86] Pouremamali F., Farhad J., Nasser S. (2020). Nrf2-ME-1 axis is associated with 5-FU resistance in gastric cancer cell line. Process. Biochem..

[87] Woo Y., Oh J., Kim J.S. (2017). Suppression of Nrf2 Activity by Chestnut Leaf Extract Increases Chemosensitivity of Breast Cancer Stem Cells to Paclitaxel. Nutrients.

[88] Chandrasekaran J., Balasubramaniam J., Sellamuthu A., Ravi A. (2021). An in vitro study on the reversal of epithelial to mesenchymal transition by brusatol and its synergistic properties in triple-negative breast cancer cells. J. Pharm. Pharmacol..

[89] Yu X., Su X., Huang X., Yao G., Song S. (2020). Brusatol: A potential anti-tumor quassinoid from Brucea javanica. Chin. Herb. Med..

[90] Rojo de la Vega M., Chapman E., Zhang D.D. (2018). NRF2 and the Hallmarks of Cancer. Cancer Cell.

[91] Torrente L., Sanchez C., Moreno R., Chowdhry S., Cabello P., Isono K., Koseki H., Honda T., Hayes J.D., Dinkova-Kostova A.T. (2017). Crosstalk between NRF2 and HIPK2 shapes cytoprotective responses. Oncogene.

[92] Zhao X.Z., Wu X.H. (2018). A small compound spindlactone A sensitizes human endometrial cancer cells to TRAIL-induced apoptosis via the inhibition of NAD(P)H dehydrogenase quinone 1. Oncol. Targets Ther..

[93] Gerard C., Goldbeter A. (2014). The balance between cell cycle arrest and cell proliferation: Control by the extracellular matrix and by contact inhibition. Interface Focus.

[94] Reddy N.M., Kleeberger S.R., Bream J.H., Fallon P.G., Kensler T.W., Yamamoto M., Reddy S.P. (2008). Genetic disruption of the Nrf2 compromises cell-cycle progression by impairing GSH-induced redox signaling. Oncogene.

[95] Marton M., Tihanyi N., Gyulavari P., Banhegyi G., Kapuy O. (2018). NRF2-regulated cell cycle arrest at early stage of oxidative stress response mechanism. PLoS ONE.

[96] Pucci B., Kasten M., Giordano A. (2000). Cell cycle and apoptosis. Neoplasia.

[97] Niture S.K., Kaspar J.W., Shen J., Jaiswal A.K. (2010). Nrf2 signaling and cell survival. Toxicol Appl. Pharmacol..

[98] Wu S., Lu H., Bai Y. (2019). Nrf2 in cancers: A double-edged sword. Cancer Med..

[99] Syu J.P., Chi J.T., Kung H.N. (2016). Nrf2 is the key to chemotherapy resistance in MCF7 breast cancer cells under hypoxia. Oncotarget.

[100] Nogueira V., Hay N. (2013). Molecular pathways: Reactive oxygen species homeostasis in cancer cells and implications for cancer therapy. Clin. Cancer Res..

[101] Sporn M.B., Liby K.T. (2012). NRF2 and cancer: The good, the bad and the importance of context. Nat. Rev. Cancer.

[102] Ham S.L., Nasrollahi S., Shah K.N., Soltisz A., Paruchuri S., Yun Y.H., Luker G.D., Bishayee A., Tavana H. (2015). Phytochemicals potently inhibit migration of metastatic breast cancer cells. Integr. Biol..

[103] Zhang H.S., Zhang Z.G., Du G.Y., Sun H.L., Liu H.Y., Zhou Z., Gou X.M., Wu X.H., Yu X.Y., Huang Y.H. (2019). Nrf2 promotes breast cancer cell migration via up-regulation of G6PD/HIF-1alpha/Notch1 axis. J. Cell Mol. Med..

[104] Nishida N., Yano H., Nishida T., Kamura T., Kojiro M. (2006). Angiogenesis in cancer. Vasc. Health Risk Manag..

[105] Grivennikov S.I., Greten F.R., Karin M. (2010). Immunity, inflammation, and cancer. Cell.

