# Supplementary material for: Targeted Inhibition of Anti-Inflammatory Regulator Nrf2 Results in Breast Cancer Retardation In Vitro and In Vivo
Source: Biomedicines. 2021 Aug 30;9(9):1119. doi: 10.3390/biomedicines9091119 (PMC8471069; doi:10.3390/biomedicines9091119)
Supplement: Supplementary file 1 [file biomedicines-09-01119-s001.zip › biomedicines-1339427-supplementary.pdf]

Supplementary Figures:

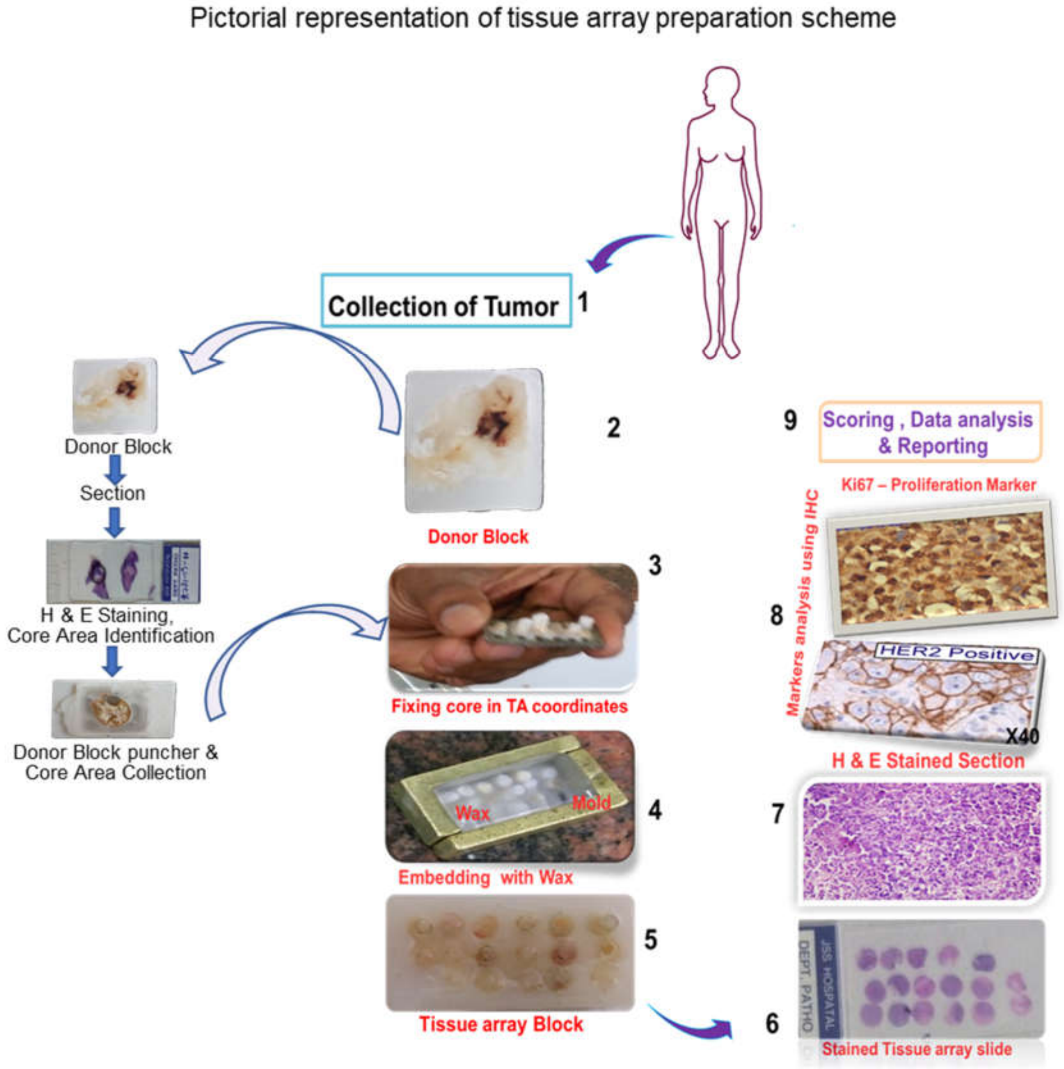

Supplemental Figure S1: ***Schematic representation of the procedure for preparing tissue array:*** Tissue arrays were prepared by first generating a donor block followed by identifying the tumor cores and fixing them in a defined pattern. The prepared array was cut in to sections of 3 to 4µm thick and stained using H & E to confirm the tumor cores. Once confirmed, the tissue arrays were used for staining using IHC protocol as detailed in materials and methods. (Figures, which are used for preparation of scheme were original)

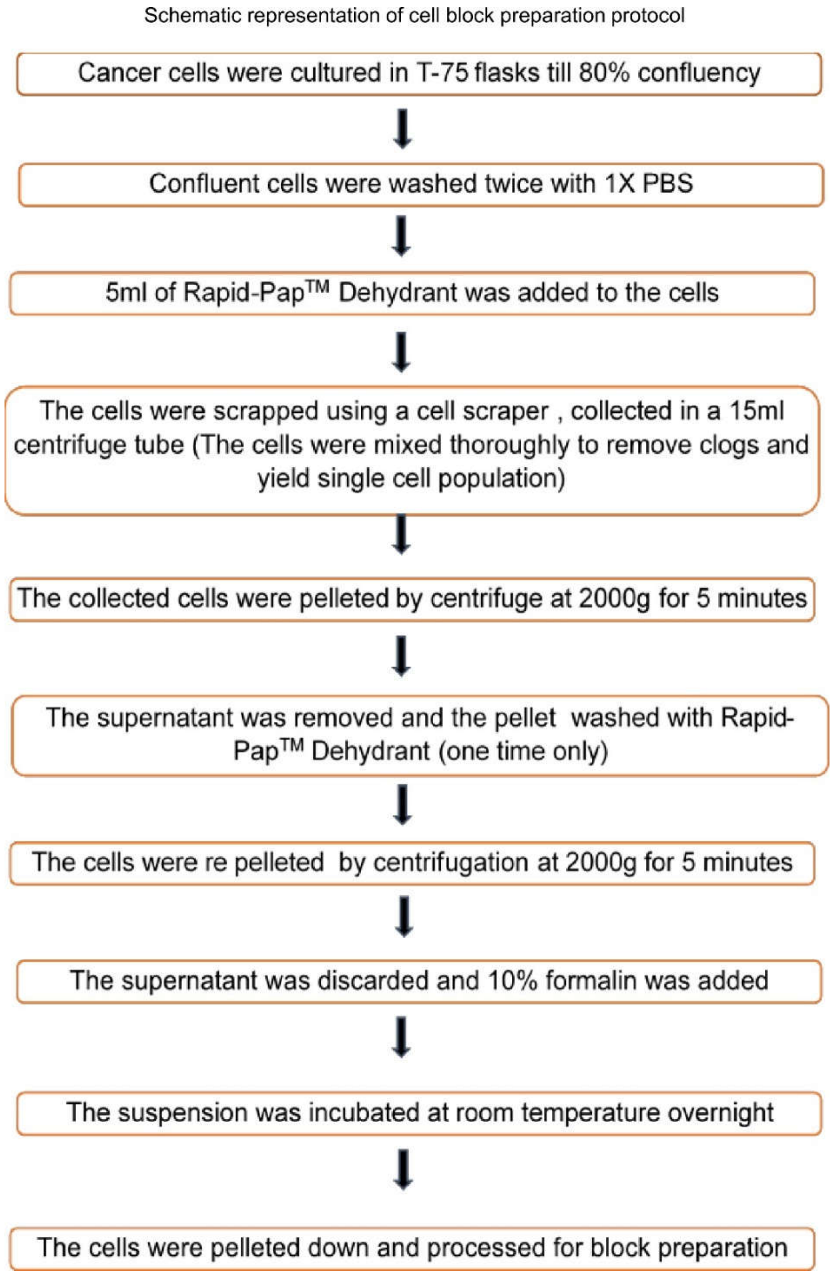

Supplemental Figure S2: ***Flow chart depicting the procedure for preparing cell blocks:*** About 10 X 10^6 cells (growing in a T-75 flask) were washed twice with 1X PBS to remove serum and other media components. Next, 5mL Rapid Pap dehydrant solution was added and cells scraped in to a 15mL conical tube and mixed thoroughly to obtain a single cell suspension. The cell suspension was centrifuged at 2000g for 5minutes and washed twice with Rapid Pap (2.0ml each time). After washing, the cell pellet was resuspended in 10% formalin and incubated at room temperature overnight. The formalin fixed cells were centrifuged, supernatant discarded. The cell pellet was used for preparing a cell block.

siRNA to Nrf2 reduced the expression at mRNA level in breast cancer cell lines

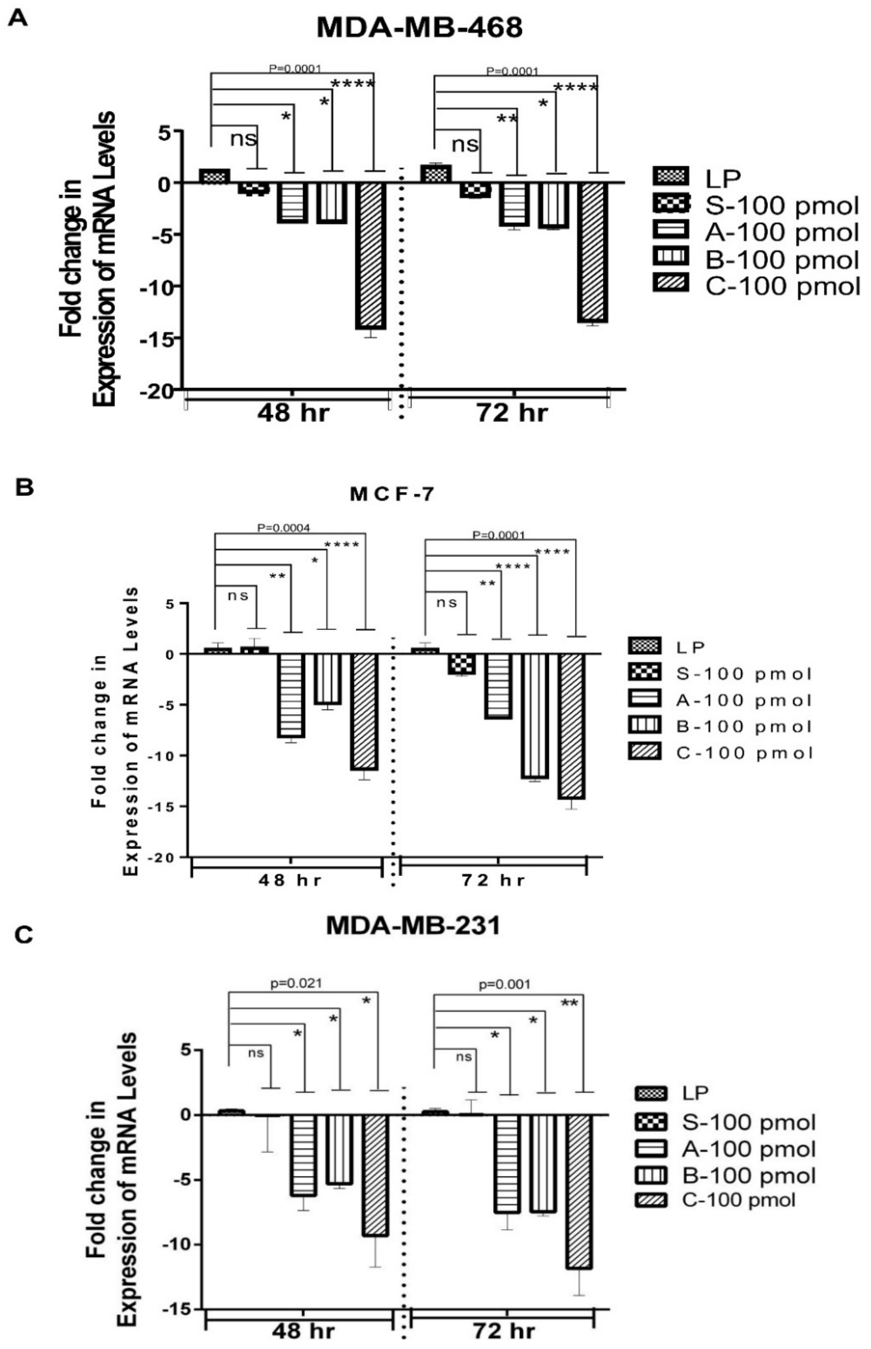

Supplemental Figure S3: ***siRNA-mediated knockdown of Nrf2 reduced it's expression at mRNA level by 4 to 14 fold in breast cancer cell lines*****:** siRNAs specifically inhibiting Nrf2 were introduced in to MDA-MB-468, MCF-7 and MDA-MB-231 cell lines using Lipofectamine RNAiMax reagent as detailed in materials and methods and total RNA isolated using Trizol (Chomczynski and Sacchi, 2006). Isolated RNA was converted in to cDNA and the expression of Nrf2 measured using realtime PCR with specific primer sets. A significant decrease in mRNA was observed at 48h and 72h post transfection. siRNA-C showed maximal inhibition (~14-fold decrease) compared to other siRNAs in all the three cell lines. One-way ANOVA test was used for the data analysis (*P<0.05, **P<0.01, ***P<0.005, ****P<0.001).

Targeting Nrf2 reduced the expression of Nrf2 protein in breast cancer cell lines

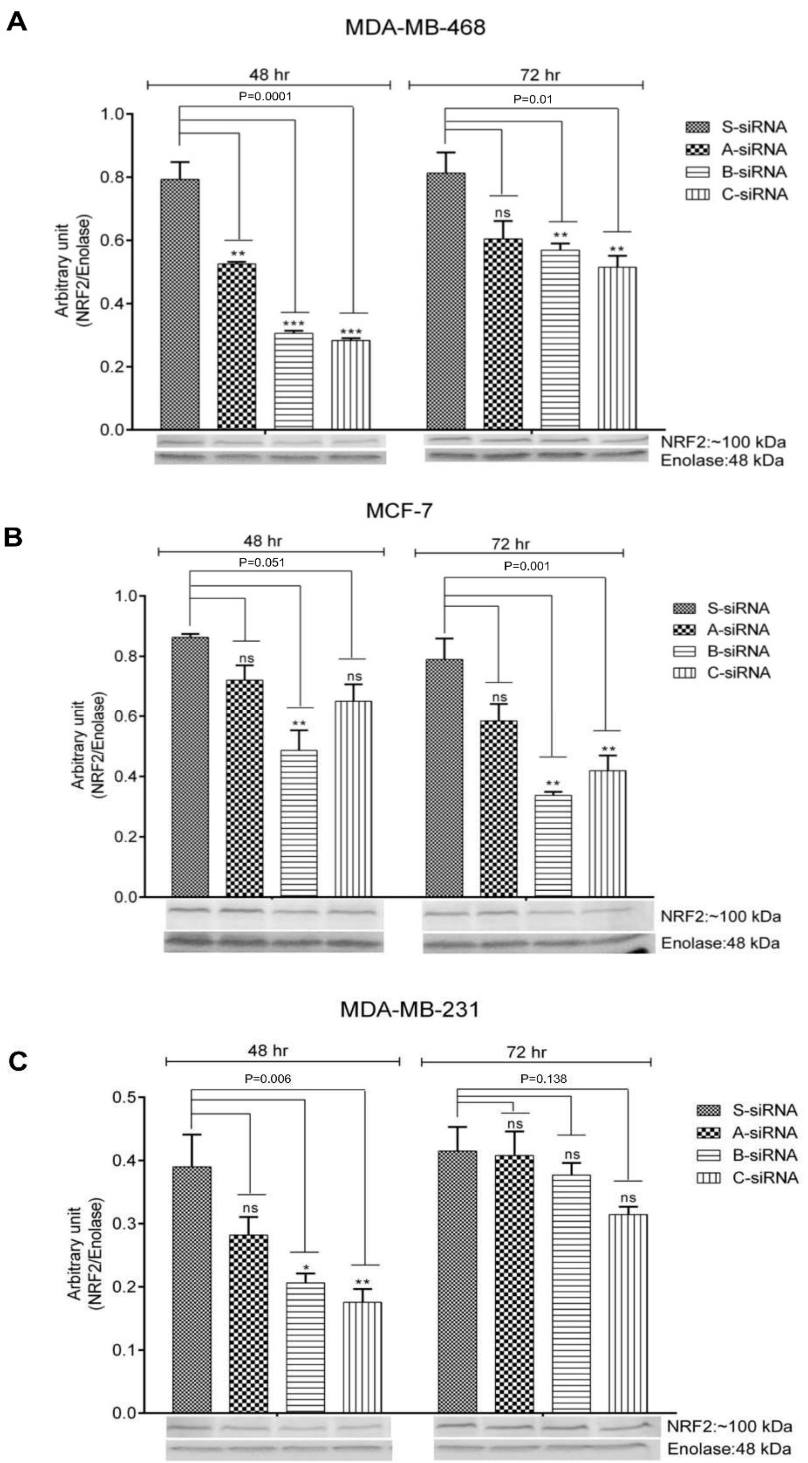

Supplemental Figure S4: ***Targeting Nrf2 using siRNA reduced the expression of Nrf2 at protein level:*** Since a 4-to-14-fold decrease in mRNA content was observed when breast cancer cells were transfected with lipofectamine RNAiMax reagent, next, the expression at protein level was assessed using western blotting. SiRNAs B and C exhibited better knockdown at 48h in all the three cell lines. However, at 72h, only SiRNA-C showed better knockdown. One-way ANOVA test was used for the data analysis (*P<0.05, **P<0.01, ***P<0.005, ****P<0.001).

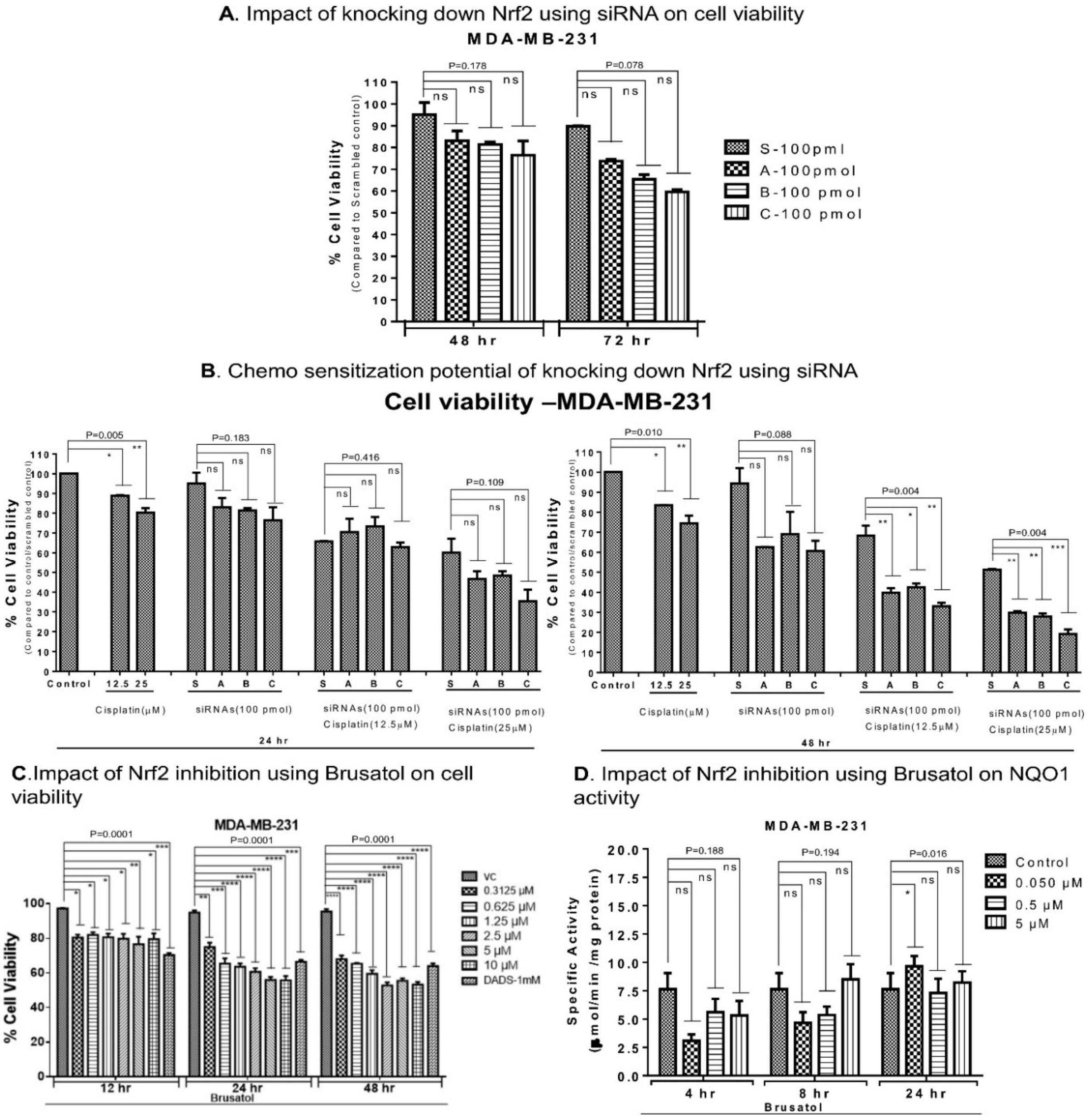

Supplemental Figure S5: ***Targeting Nrf2 using siRNAs reduced the viability of MDA-MB-231 breast cancer cells in vitro.*** In order to determine whether targeted inhibition of Nrf2 reduces the viability of breast cancer cell line, 100picomoles of siRNA was introduced in to MDA-MB-231(5A) cell lines and viability determined using MTS after 48h and 72h of transfection. A non-significant 10% to 30% reduction in cell growth was observed with siRNA-B and siRNA-C. ***siRNA mediated Nrf2 inhibition sensitized breast cancer cells MDA-MB-231 to chemotherapeutic drug cisplatin*****:** A significant decrease in MDA-MB-231 cell growth was observed when Nrf2 inhibition was combined with cisplatin treatment (5B) in MDA-MB-231 (5B; P value >0.05; by One-way ANOVA). ***Pharmacological inhibition of Nrf2 using brusatol retarded breast cancer cells proliferation in vitro*****:** Treatment of breast cancer cell line MDA-MB-231 with increasing concentrations (0.3125µM to 10µM) of brusatol for 12h, 24h and 48h retarded cell growth in a time dependent manner (5C). Under these treatment conditions, a non-significant decrease in NQO1 activity was observed (5D). One-way ANOVA test was used for the data analysis (*P<0.05, **P<0.01, ***P<0.005, ****P<0.001).

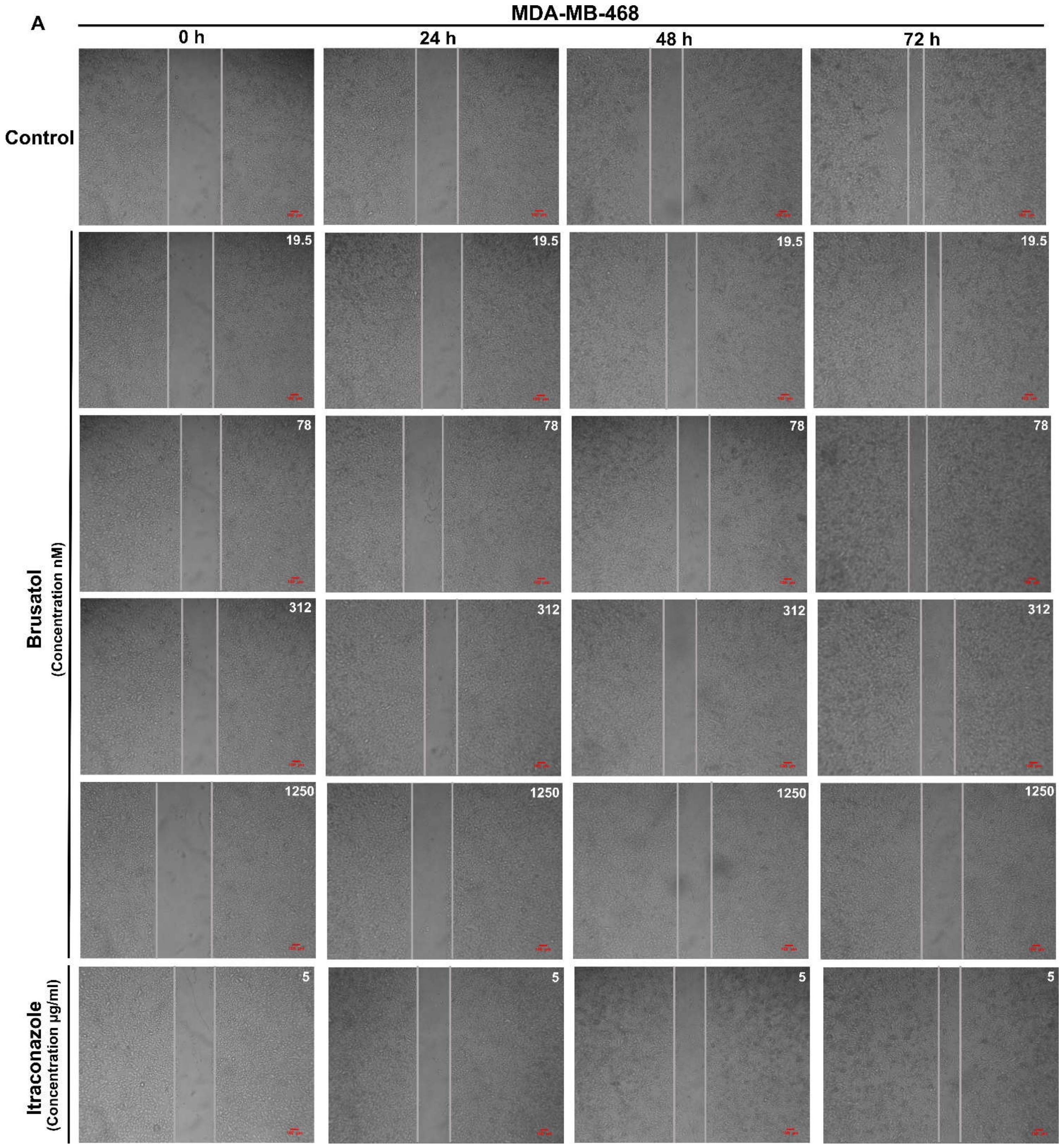
A
MDA-MB-468
0 h
24 h
48 h
72 h
Control
Brusatol
(Concentration nM)
19.5
78
312
1250
Itraconazole
(Concentration µg/ml)
5

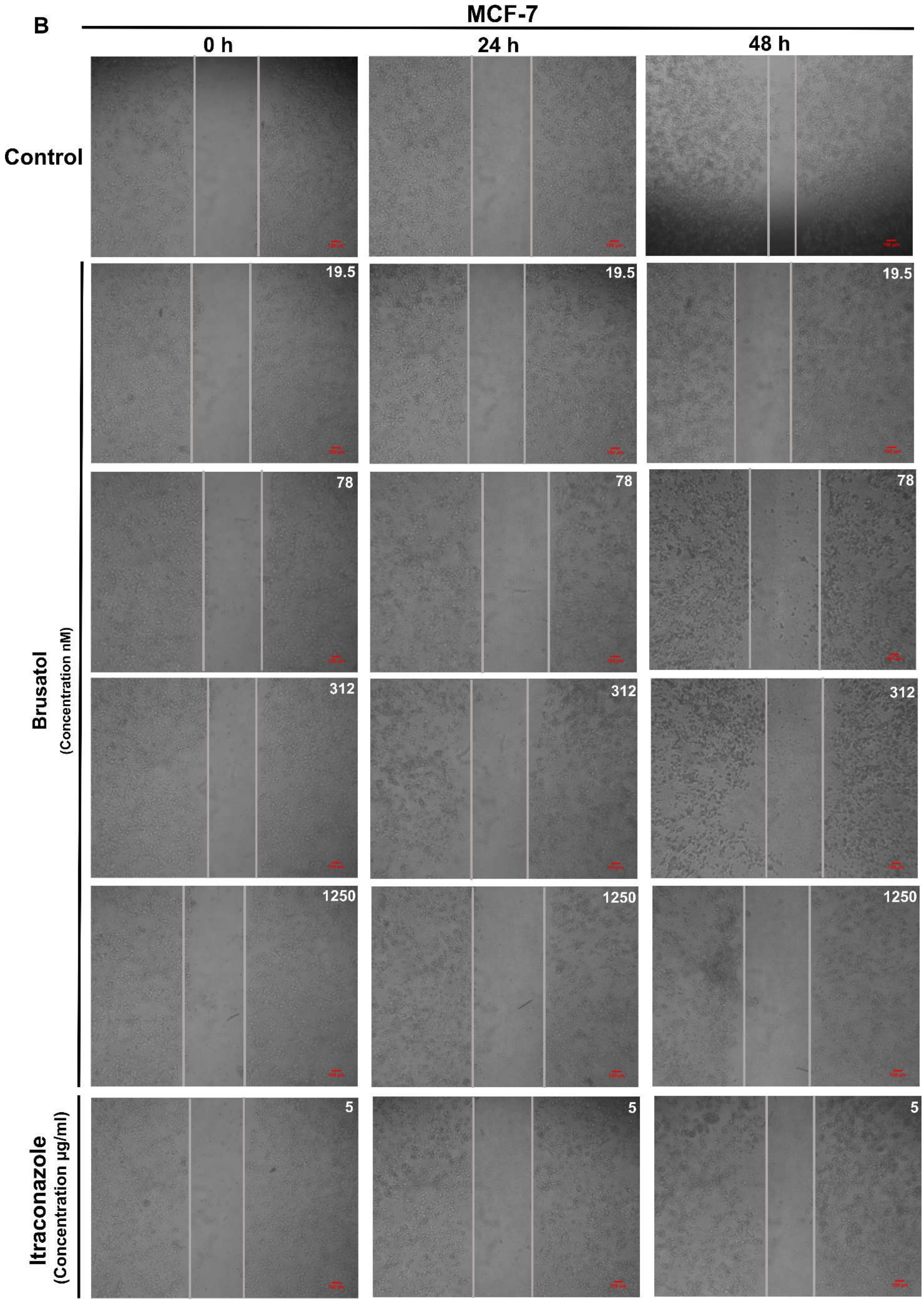

B
MCF-7
0 h
24 h
48 h
Control
Brusatol
(Concentration nM)
19.5
78
312
1250
Itraconazole
(Concentration µg/ml)
5

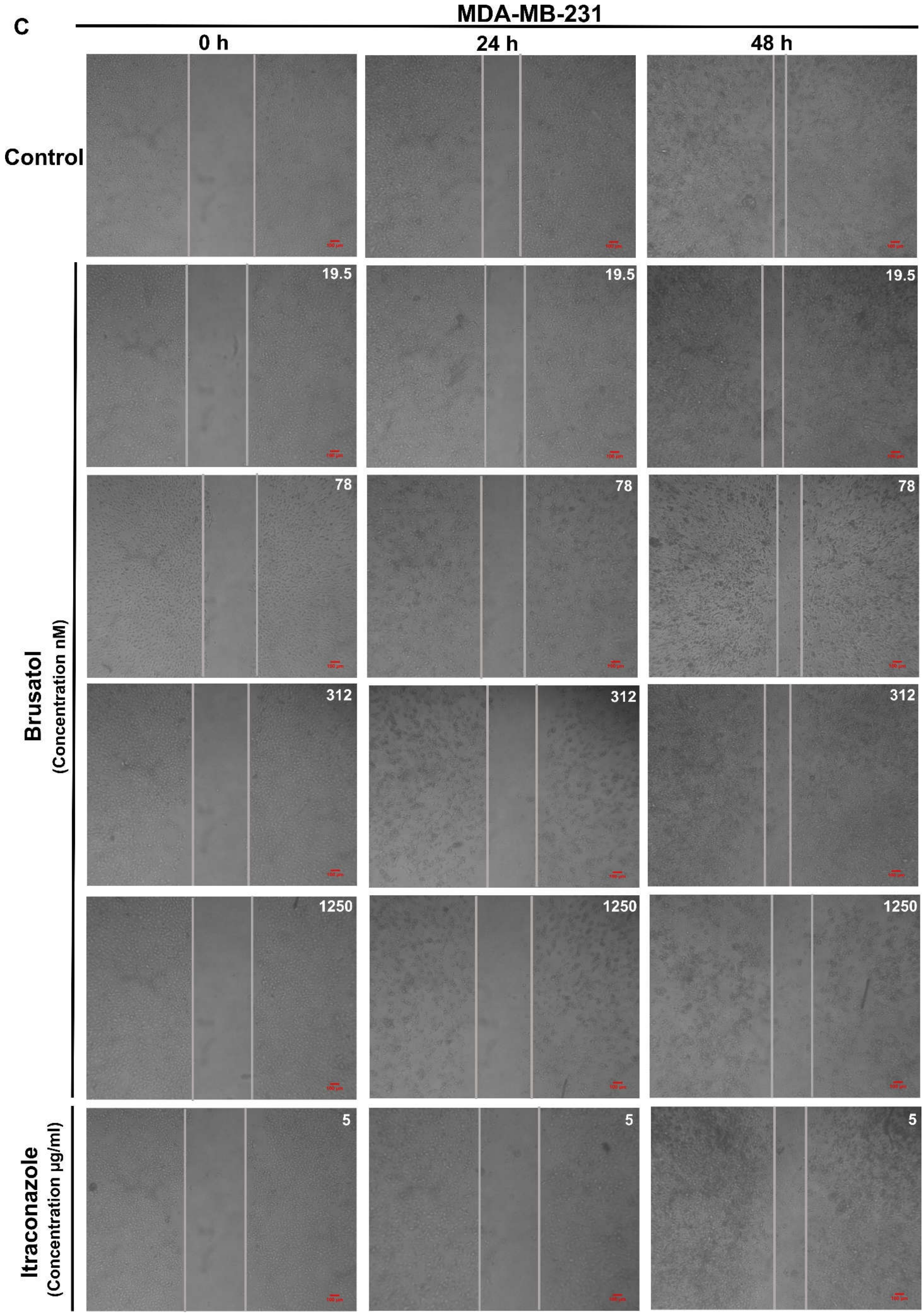
C
MDA-MB-231
0 h
24 h
48 h
Control
Brusatol
(Concentration nM)
19.5
78
312
1250
Itraconazole
(Concentration µg/ml)
5

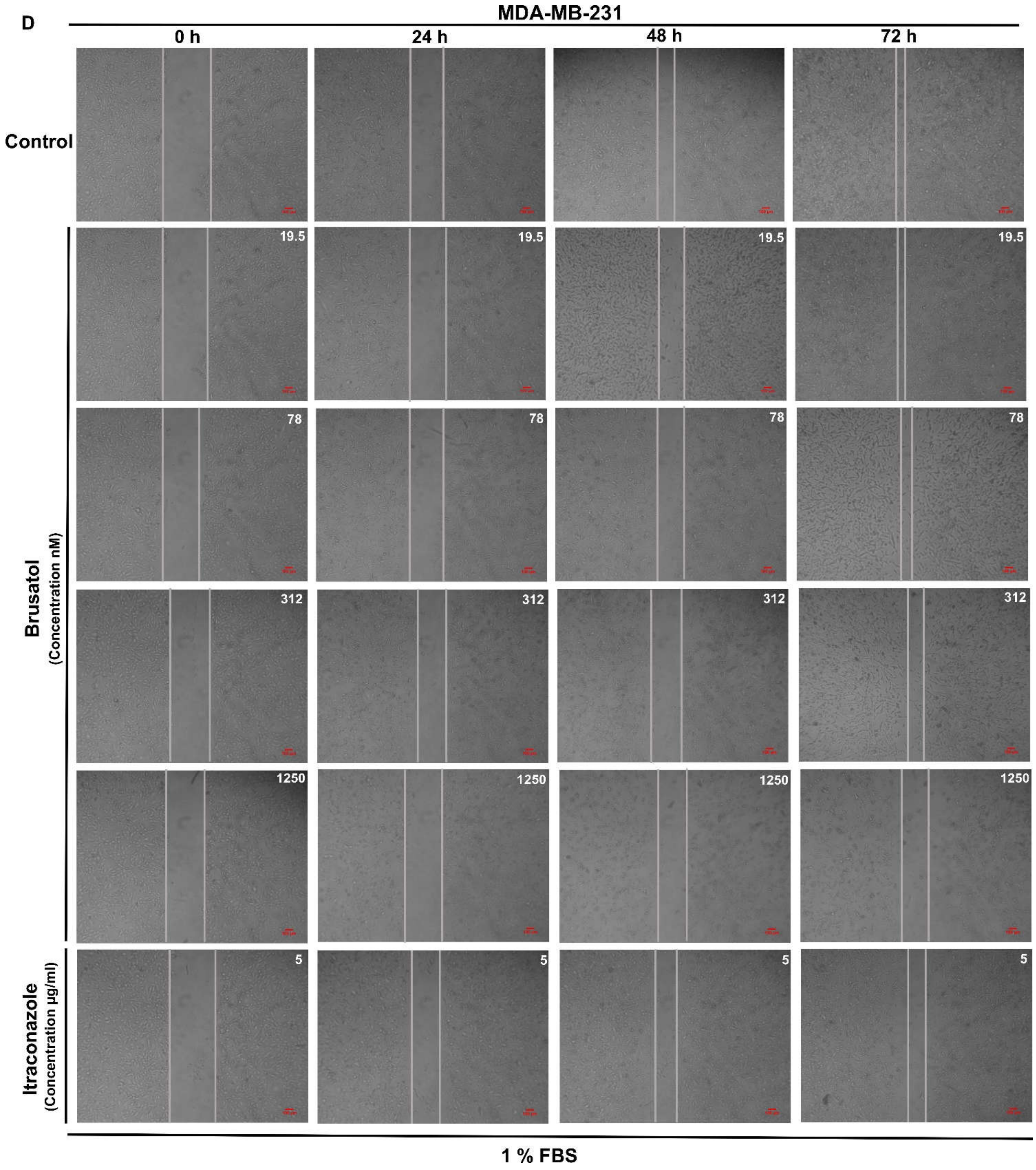

Supplemental Figure S6: *Pharmacological inhibition of Nrf2 using brusatol retarded cell migration in breast cancer cells*: The effect of brusatol on cell migration was studied by incubating MDA-MB-468 (6A), MCF-7 (6B), MDA-MB-231 (6C) and MDA-MB-231 cells growing in 1% of FBS (6D) with non-toxic concentration of brusatol, followed by measuring the area left after 24h, 48h and 72h of exposure. Brusatol treatment inhibited the migration of breast cancer cells, in particular the MCF-7 cell line (Scale:100µm).

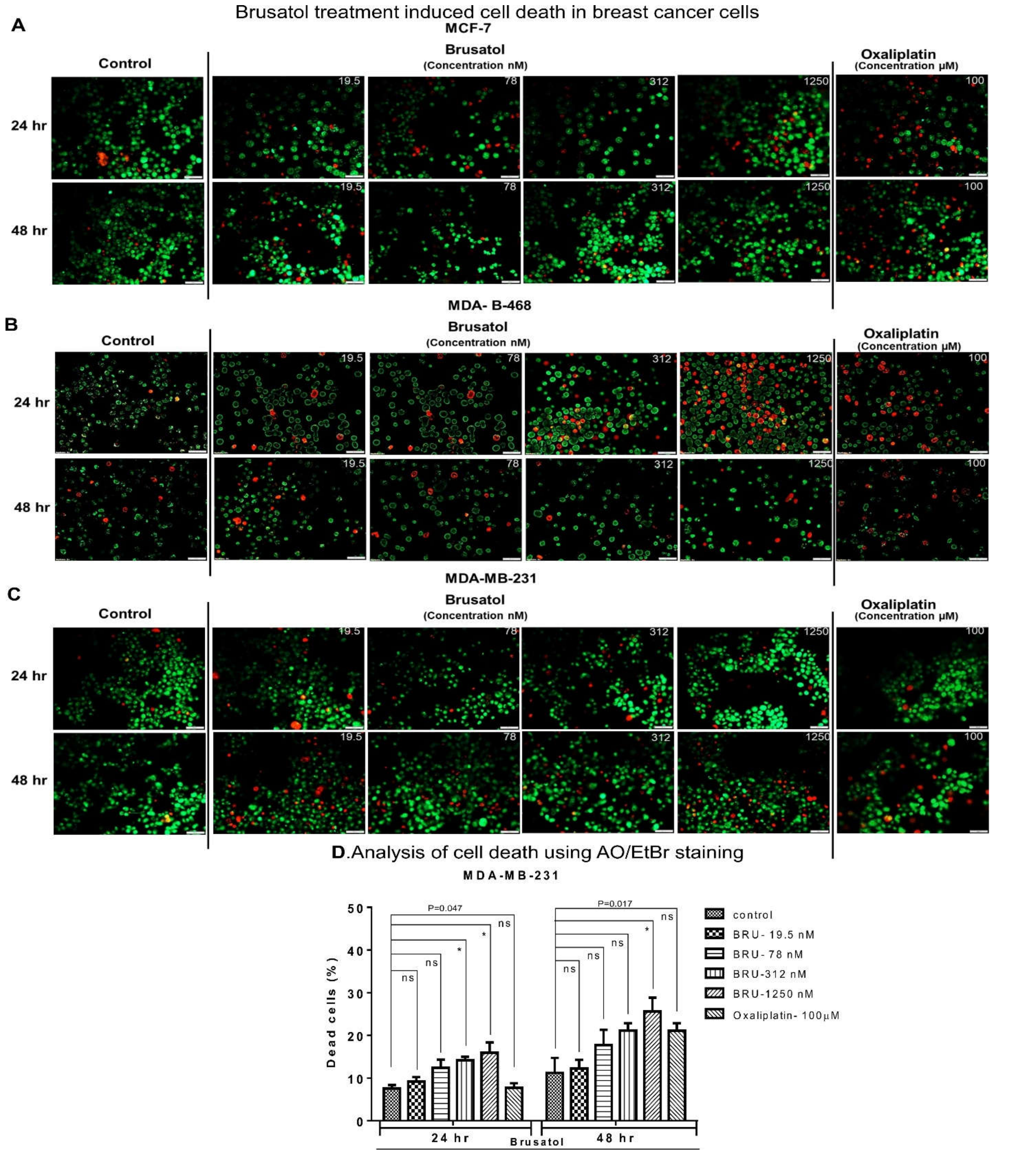

Supplemental Figure S7: ***Treatment with brusatol induced death in breast cancer cells*:** In order to determine the impact of treatment of breast cancer cells with brusatol on cell survival and death, the control and treated cells were stained with acridine orange and ethidium bromide and live (Green colored), apoptotic (Orange colored) and dead (Red colored) cells counted. A significant increase in dead cells was observed upon treatment of MCF-7 (7A), MDA-MB-468 (7B) and MDA-MB-231 (7C &7D) with increasing concentrations (19.5nM to 1250nM) brusatol for 24h and 48h. Oxaliplatin was used as positive control. One-way ANOVA test was used for the data analysis (*P<0.05, **P<0.01, ***P<0.005, ****P<0.001).

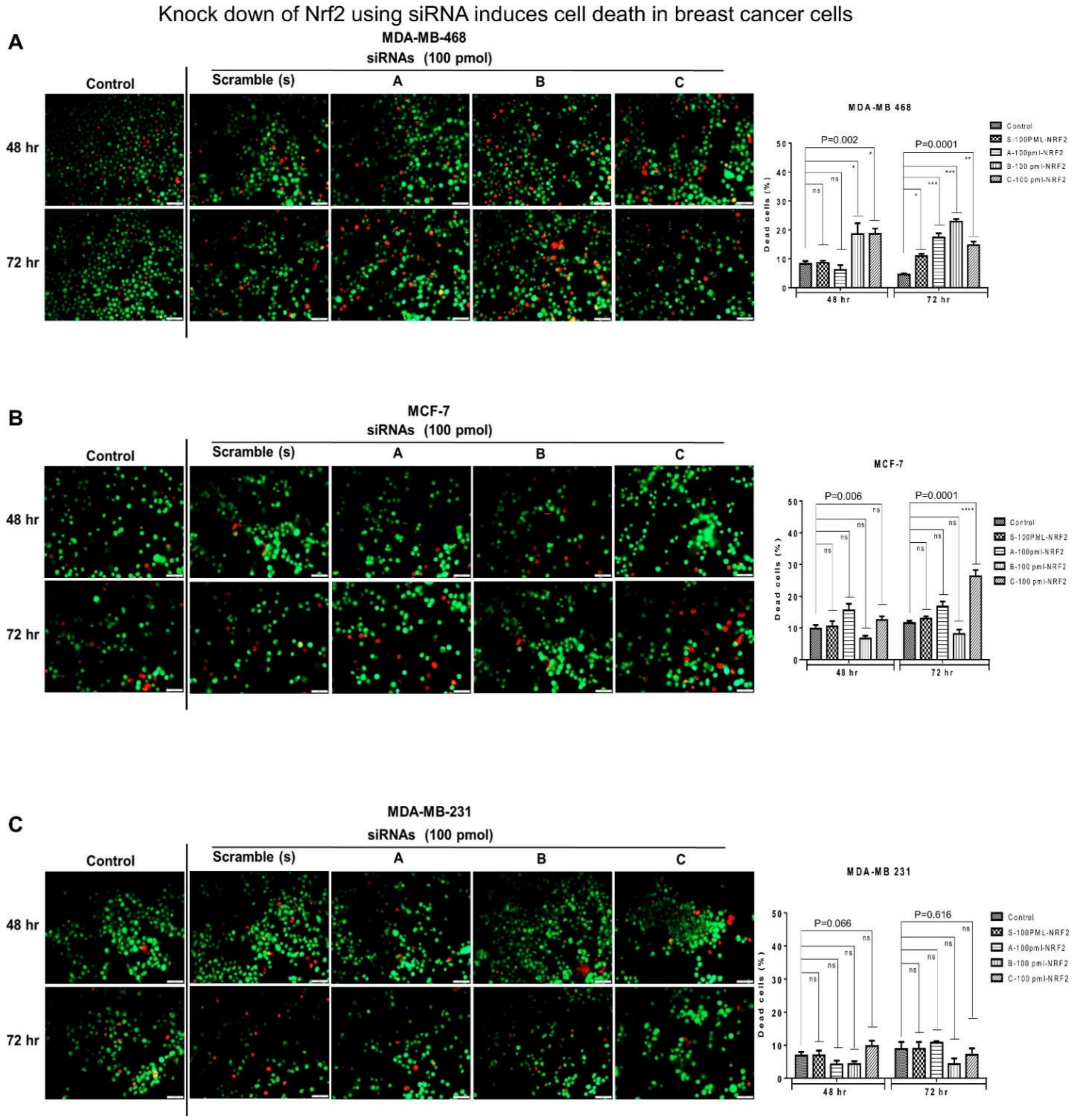

Supplemental Figure S8: ***Targeted inhibition of Nrf2 using siRNA induced cell death in breast cancer cells***: To assess the impact of targeting Nrf2 using siRNA, breast cancer cell lines MDA-MB-468(8A), MCF-7(8B) and MDA-MB-231 (8C) were transfected with 100picomoles of siRNA to Nrf2 and after 48h and 72h analyzed using acridine orange and ethidium bromide staining as detailed in materials and methods. Number of live (Green colored), apoptotic (Yellow colored) and dead (Red colored) cells were counted and percentages calculated. Analysis of the data showed a significant increase in dead cells upon transfecting MDA-MB-468 with siRNA to Nrf2. The impact is more at 72h post transfection (8A). In case of MCF-7, although an increase in dead cell population was observed with siNrf2-A, the impact is more with siNrf2-C at 72h (8B). Interestingly, knocking down Nrf2 had not increased the dead cell population in MDA-MB-231 cell line (8C). One-way ANOVA test was used for the data analysis (*P<0.05, **P<0.01, ***P<0.005, ****P<0.001).

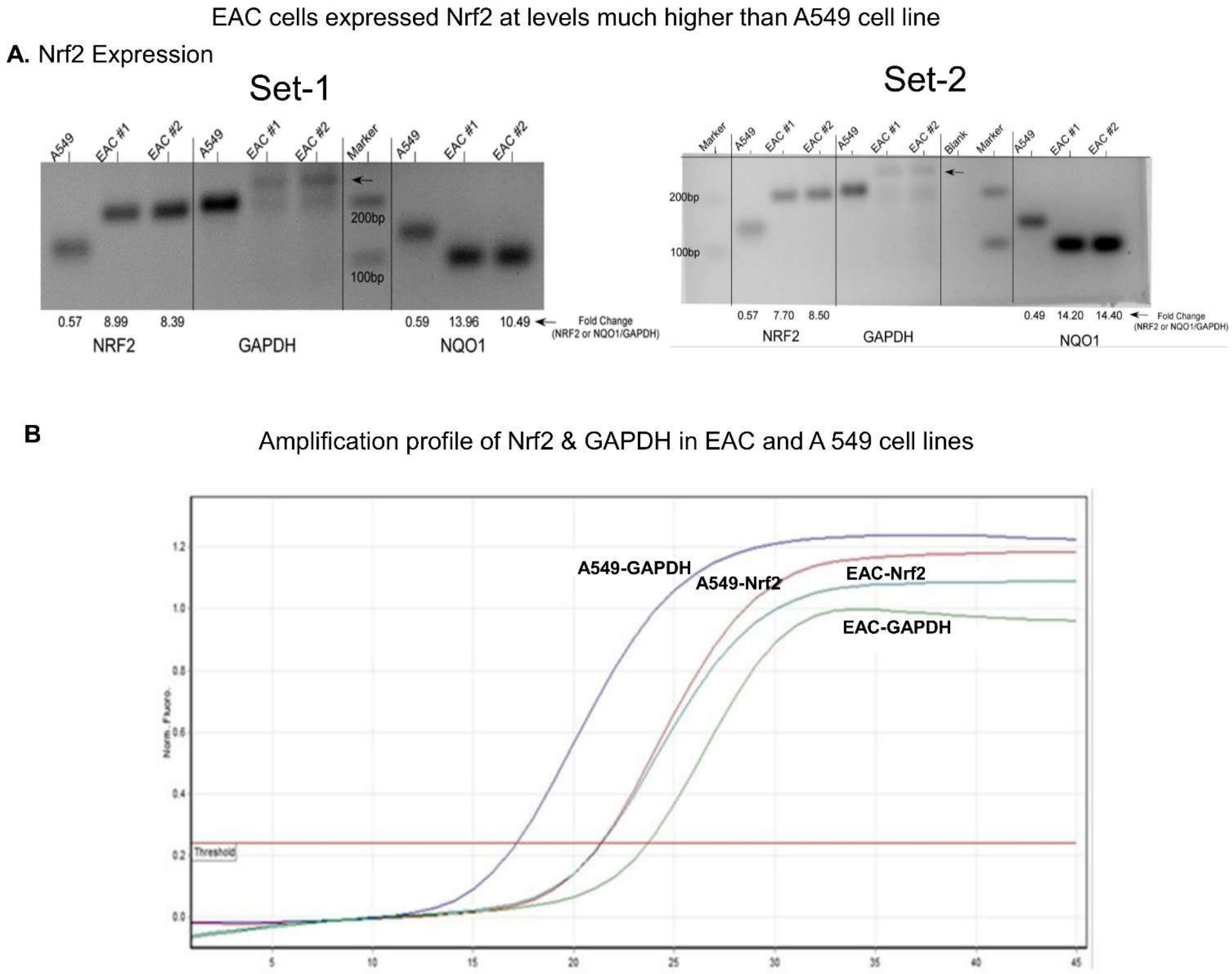

Supplemental Figure S9: ***EAC cells expressed elevated Nrf2 compared to positive control A549 cell line*****:** To check the efficacy of Nrf2 inhibitor Brusatol in EAC model, first, the expression level of Nrf2 was determined in the EAC cells using qRT-PCR as detailed in materials and methods. Interestingly, the expression of Nrf2 is at least 14-fold higher compared to the positive control cell line A549 (9A). Even the target gene NQO1 is also much higher in EAC cells indicating the suitability of EAC cells for tumor kinetics study. The amplification profile showed similar Ct values for Nrf2 in A549 and EAC, however, the Ct value of GAPDH was much higher for EAC compared to A549 (9B).

A Schematic representation of the dose and parameter measurement schedules in mice

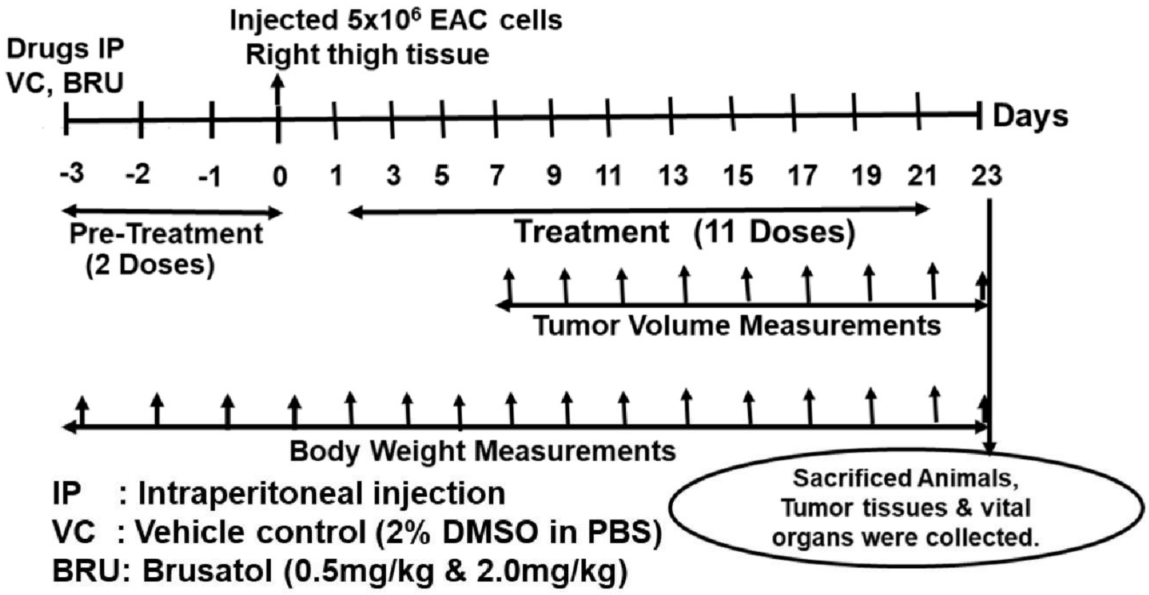

B Photos demonstrating tumor bearing mice in each group

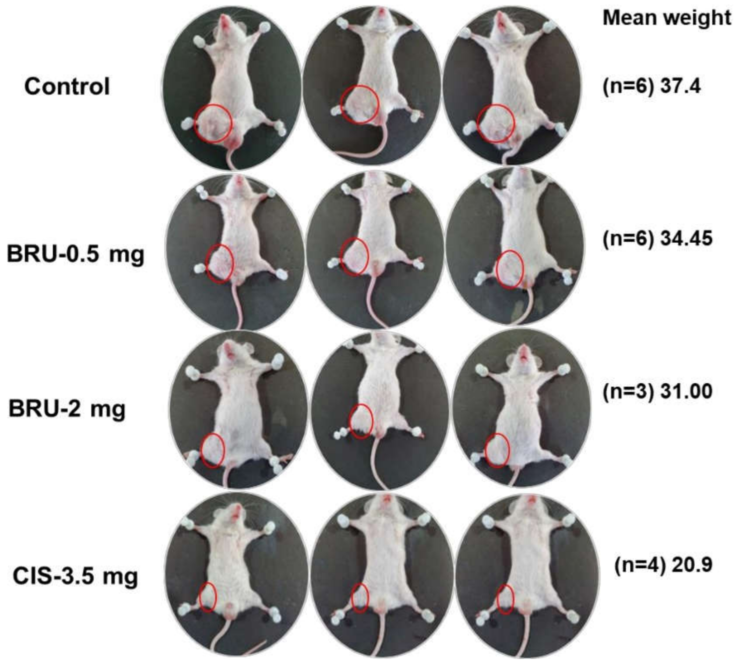

Supplemental Figure S10: ***Schematic representation of in vivo study protocol and the photographs of mice***: The impact of intraperitoneal administration of brusatol on EAC solid tumors growth was studied by following the scheme shown in Supplemental Figure 10A. In brief, 2 doses (0.5mg/kg and 2.0mg/kg) of brusatol and vehicle control were administered intraperitoneally, even before injecting 5 X 10^6 EAC cells in the thigh region. After cell injection, the mice were treated with 0.5mg/kg and 2.0mg/kg body weight brusatol every other day for a period of 20 days. During the experiment, the body weight, tumor volume were measured. At the end of the experiment, mice were photographed (10B) tumors harvested and weighed. The collected tumors were processed for lysate collection as well as immunohistochemical staining.

Supplemental Tables:

Supplemental Table S1: Immunohistochemical (IHC) analysis of normal breast tissues for Nrf2 expression

Nrf2 Expression in Normal Breast Tissues			
Intensity of staining		% of Cells Stained	
Cytoplasm	Nucleus	Cytoplasm	Nucleus
1	0	2	1
1	0	2	0
1	0	3	1

Immunohistochemical (IHC) analysis of grade-II and grade-III breast cancer tissues for Nrf2, ER, PR and HER2

Nrf2 Expression in Malignant tumors								
Tumor Number	Intensity of Staining		% Cells Stained			Receptor Status		
	Cytoplasm	Nucleus	Cytoplasm	Nucleus	Tumor Grade	ER	PR	HER2
1	1	1	10	10	2	0	0	0
2	2	2	60	66	2	0	0	0
3	2	2	33	30	2	0	0	0
4	1	1	10	32	2	1	1	0
5	1	1	1	10	2	1	1	0
6	1	1	1	1	2	1	1	1
7	1	1	10	10	2	1	1	1
8	1	0	1	0	2	1	1	0
9	1	0	1	0	2	1	1	1
10	1	0	1	0	2	1	1	1
11	1	1	1	10	2	1	1	1
12	1	0	30	0	2	0	0	1
13	1	0	10	0	2	1	1	1
14	1	0	90	0	2	0	0	0
15	1	2	20	10	3	0	0	0
16	0	1	0	1	3	1	1	1
17	0	0	0	0	3	1	1	1
18	1	1	1	30	3	0	0	1
19	1	0	65	0	3	1	1	0
20	0	1	0	1	3	0	0	1
21	1	2	30	60	3	1	1	1

22	1	1	10	1	3	1	1	0
23	0	1	0	5	3	1	1	0
24	1	2	33	0	3	1	1	0
25	0	0	0	0	3	0	0	1
26	1	1	10	10	3	1	1	1
27	1	0	1	0	3	1	1	1
28	0	0	0	0	3	1	1	1
29	1	0	1	0	3	0	0	0
30	1	1	1	1	3	1	1	1
31	1	1	10	1	3	1	1	0
32	0	0	0	0	3	1	1	0
33	1	1	1	1	3	0	0	1
34	1	1	1	1	3	1	1	1
35	1	0	0	10	3	1	0	1
36	2	1	65	60	3	1	1	1
37	2	1	65	60	3	1	1	1
38	1	1	33	33	3	0	0	1
39	1	1	90	66	3	1	1	0
40	1	1	1	1	3	1	1	1
41	1	0	1	0	3	1	1	0
42	1	1	1	1	3	1	1	1
43	1	1	1	1	3	0	0	0

Supplemental Table S1: Immunohistochemical staining scores of normal, grade-II and grade-III breast cancer tissues: Normal tissues as well as breast cancer tissues (Grade-II and Grade-III) were processed as detailed in materials and methods and immunohistochemical staining was performed to detect Nrf2, ER, PR and HER2. The staining intensity and percentage cells stained both in cytosolic and nuclear regions were scored and represented in the table. Very minimal staining intensity and percentage cells stained was observed in normal tissues. The grade-II and grade-III tumors had much higher expression of Nrf2 compared to normal tissues. Among 14 grade-II tumors, 4 were triple negative and had high expression of Nrf2 (in cytosol or nucleus or both regions). Among 29 grade-III tumors, only 3 found to be triple negative tumor, and only one tumor had ~20% (cytosol region) and 10% (nuclear region) cells stained for Nrf2, with a staining intensity score of 1 and 2, respectively in cytosol and nuclear regions.

Supplemental Table S2: Nucleotide sequence of siRNAs

siNrf2				
siRNA	siRNA ID	siRNA Sequence	Binding site (NCBI Reference Sequence)	siRNA Location
A	HSS181506	CAATGAAGCTCAACTTGCATTAATT	NM_001145413.3	2143
B	HSS181505	CAAACTGACAGAAGTTGACAATTAT	NM_001145412.3	1348
C	HSS107130	CCAACCAGTTGACAGTGAACTCATT	NM_001145412.3	1482

Table S2: Nucleotide sequence of siRNAs used in this study: Three independent validated stealth siRNAs targeting Nrf2 have been procured from Life Technologies Inc, USA, and used for knocking down Nrf2 in breast cancer cell lines. The table describes the nucleotide sequence, siRNA location and the binding site reference sequence Id from NCBI.

Supplemental Table S3: The clinical features of the grade-II and grade-III tumors and keys to master chart.

Grade -II Master chart																					
Sno	TMA-SN	AGE	MC	MP	AAFCB	SIDE	SITE	LI	D	NC	LVI	PNI	G	LST	T	N	ER	PR	HER	MC	NPG
1	5	37	11	0	2	1	3	0	1	1	1	0	2	1.8	1	0	0	0	0	3	1
2	9	60	11	1	1	2	5	0	1	0	0	0	2	3	2	1	0	0	0	3	3
3	10	52	12	1	1	2	1	0	1	1	1	0	2	5	2	1	0	0	0	3	3
4	12	48	12	0	2	2	2	0	1	0	1	1	2	1.8	1	1	1	1	0	1	2
5	13	48	12	0	1	2	1	1	1	0	1	0	2	3.6	2	1	1	1	0	1	3
6	3	63	11	1	1	1	4	1	1	0	1	1	2	3	2	1	1	1	1	2	3
7	4	45	12	0	1	2	3	1	0	0	1	1	2	2	1	1	1	1	1	2	2
8	7	50	13	1	3	1	1	0	0	1	0	0	2	2.5	2	0	1	1	0	1	2
9	9	48	12	0	2	2	1	0	0	1	0	0	2	4.5	2	0	1	1	1	2	2
10	11	46	13	0	1	2	1	1	0	1	1	1	2	2	1	3	1	1	1	2	4
11	16	38	11	0	2	2	1	1	1	0	0	1	2	4	2	1	1	1	1	2	3
12	17	63	12	1	2	1	3	0	0	1	1	0	2	3.5	2	2	0	0	1	4	5
13	8	40	12	0	2	1	1	0	1	0	1	0	2	6	3	2	1	1	1	2	5
14	16	60	12	1	0	2	5	1	1	1	1	0	2	3.5	2	1	0	0	0	3	4

Grade -III Master chart

Sno	TMA-SN	AGE	MC	MP	AAFCB	SIDE	SITE	LI	D	NC	LVI	PNI	G	LST	T	N	ER	PR	HER	MC	NPG
1	3	60	12	1	1	1	1	1	1	1	1	0	3	3.5	2	2	0	0	0	3	5
2	6	50	13	1	1	1	1	0	1	0	1	1	3	5.5	3	2	1	1	1	2	5
3	7	55	14	1	0	2	1	1	1	0	1	0	3	5.5	3	3	1	1	1	2	5
4	8	50	11	1	1	2	4	1	1	1	1	0	3	2.2	2	2	0	0	1	4	4
5	11	70	11	1	0	2	3	0	1	0	1	0	3	3	2	1	1	1	0	1	4
6	14	50	12	1	2	1	4	1	1	1	1	0	3	5.4	3	0	0	0	1	4	3
7	16	35	12	0	1	2	5	0	1	1	1	1	3	3	2	2	1	1	1	2	5
8	15	40	11	0	0	2	1	0	1	1	1	0	3	1.8	1	2	1	1	0	1	4
9	17	65	11	1	1	2	3	1	1	0	1	0	3	2	1	1	1	1	0	1	3
10	18	52	11	1	1	2	3	1	0	1	0	0	3	5.5	3	0	1	1	0	1	2
11	5	74	12	1	1	1	1	1	1	1	1	0	3	5.8	3	2	0	0	1	4	5
12	6	45	12	0	1	2	4	0	1	1	1	1	3	6	3	2	1	1	1	2	5
13	8	60	11	1	2	1	3	1	0	1	0	0	3	3.5	2	2	1	1	1	2	5
14	10	55	13	1	1	1	2	0	1	1	1	0	3	5	2	3	1	1	1	2	5
15	13	63	13	1	0	1	3	1	1	1	1	0	3	2.5	2	3	0	0	0	3	5
16	14	60	12	1	1	1	5	1	1	0	1	0	3	5.5	3	3	1	1	1	2	5
17	15	46	11	0	2	1	1	0	1	0	0	1	3	3	2	0	1	1	0	1	2
18	18	51	12	1	1	2	1	0	1	1	0	0	3	5.4	3	1	1	1	0	1	4
19	3	45	13	0	2	1	1	1	1	1	1	0	3	6	3	2	0	0	1	3	5
20	6	73	11	1	3	2	5	1	1	0	1	0	3	5	2	2	1	1	1	2	5
21	7	55	11	1	2	1	5	1	1	0	1	0	3	2.5	2	2	1	0	1	2	4
22	9	60	11	1	1	2	5	1	1	0	1	0	3	3.5	2	0	1	1	1	2	3
23	10	53	12	1	1	1	5	1	1	1	0	0	3	5	2	0	1	1	1	2	3
24	11	38	11	0	1	2	2	0	1	0	1	0	3	3	2	0	0	0	1	4	2
25	12	37	11	0	2	1	1	1	1	1	1	0	3	3	2	1	1	1	0	1	4
26	13	52	11	1	3	1	5	1	1	0	1	0	3	8	3	2	1	1	1	2	5

27	14	55	11	1	2	1	1	0	1	0	1	0	3	4	2	3	1	1	0	1	4
28	17	60	12	1	1	1	1	1	1	0	1	0	3	3	2	0	1	1	1	2	3
29	18	45	12	0	1	2	1	1	1	1	1	0	3	3.5	2	0	0	0	0	3	3

KEYS TO MASTER CHART	
**SN**	Serial number
**MC**	Menarche
**MP**	Menopause
**0**	Pre-menopause
**1**	Post-menopause
**AAFCB**	Age at 1st child birth
**0**	Nulliparous
**1**	15-19 years
**2**	20-24 years
**3**	25-29 years
**SIDE**	Side of the tumor
**1**	Right
**2**	Left
**SITE**	Location of the tumor
**1**	Upper outer quadrant
**2**	Upper inner quadrant
**3**	Lower outer quadrant
**4**	Lower inner quadrant
**5**	Nipple areola complex
**LI**	Lymphocytic infiltration
**0**	NO
**1**	Yes
**D**	Desmoplasia
**0**	NO
**1**	Yes
**NC**	Necrosis
**0**	NO
**1**	Yes
**Lvi**	Lymphovascular invasion
**0**	NO
**1**	Yes
**PNI**	Perineuronal invasion
**0**	NO
**1**	Yes
**G**	Histopathological grading
**1**	Grade 1
**2**	Grade 2
**3**	Grade 3

**LST**	Largest size of tumor in centimeter
**p**	Pathologic tumor size (pT)
**1**	T1
**2**	T2
**3**	T3
**4**	T4
**N**	Lymphnode status
**0**	N0
**1**	N1
**2**	N2
**3**	N3
**ER**	Estrogen receptor
**0**	Negative
**1**	Positive
**PR**	Progesteron receptor
**0**	Negative
**1**	Positive
**HER**	Human epidermal growth factor receptor
**0**	Negative
**1**	Positive
**MC**	Molecular classification
**1**	Luminal A
**2**	Luminal B
**3**	Triple negative
**4**	HER2/new positive
**NPG**	Nottingham Prognostic Groups
**1**	Good Prognostic Group
**2**	Moderate Prognostic Group 1
**3**	Moderate Prognostic Group II
**4**	Poor Prognostic Group
**5**	Very poor Prognostic Group
**CD10**	CD10 Score
**0**	Negative
**1**	Weakly positive
**2**	Strongly positive
